# Chromatin remodelling and antisense-mediated up-regulation of the developmental switch gene *eud-1* control predatory feeding plasticity

**DOI:** 10.1038/ncomms12337

**Published:** 2016-08-04

**Authors:** Vahan Serobyan, Hua Xiao, Suryesh Namdeo, Christian Rödelsperger, Bogdan Sieriebriennikov, Hanh Witte, Waltraud Röseler, Ralf J. Sommer

**Affiliations:** 1 Department for Evolutionary Biology, Max-Planck-Institute for Developmental Biology, Spemannstrasse 35, 72076 Tübingen, Germany

## Abstract

Phenotypic plasticity has been suggested to act through developmental switches, but little is known about associated molecular mechanisms. In the nematode *Pristionchus pacificus*, the sulfatase *eud-1* was identified as part of a developmental switch controlling mouth-form plasticity governing a predatory versus bacteriovorous mouth-form decision. Here we show that mutations in the conserved histone-acetyltransferase *Ppa-lsy-12* and the methyl-binding-protein *Ppa-mbd-2* mimic the *eud-1* phenotype, resulting in the absence of one mouth-form. Mutations in both genes cause histone modification defects and reduced *eud-1* expression. Surprisingly, *Ppa-lsy-12* mutants also result in the down-regulation of an antisense-*eud-1* RNA. *eud-1* and antisense-*eud-1* are co-expressed and further experiments suggest that antisense-*eud-1* acts through *eud-1* itself. Indeed, overexpression of the antisense-*eud-1* RNA increases the *eud-1*-sensitive mouth-form and extends *eud-1* expression. In contrast, this effect is absent in *eud-1* mutants indicating that antisense-*eud-1* positively regulates *eud-1*. Thus, chromatin remodelling and antisense-mediated up-regulation of *eud-1* control feeding plasticity in *Pristionchus*.

## Abstract (web summary)

In the nematode *Pristionchus pacificus*, a developmental switch, the sulfatase *eud-1*, controls mouth-form plasticity. Here, the authors show that mutations in two conserved histone modifying enzymes mimic the *eud-1* phenotype, in part mediated by an antisense *eud-1* RNA, resulting in the absence of one mouth-form

## Graphical abstract

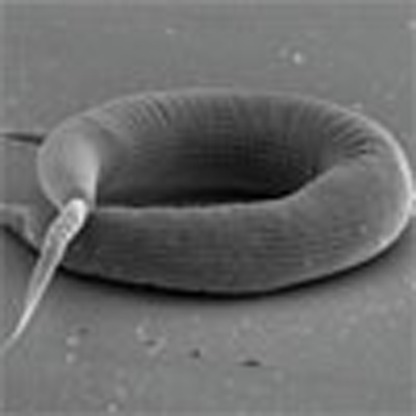

Developmental (phenotypic) plasticity has been suggested to facilitate morphological novelty and diversity^1^,^2^,^3^,^4^,^5^, but little is known about the molecular aspects of developmental switch mechanisms that underlie plasticity. The nematode *Pristionchus pacificus* is a potential model system to study the molecular and mechanistic details of developmental plasticity because it can be easily cultured in the laboratory by feeding on bacteria, but in the wild it lives in a necromenic interaction with beetles^6^,^7^. Specifically, the necromenic life style of *P. pacificus* and related nematodes is facilitated by dynamic feeding mode switching between bacterial grazing and the predation of other nematodes (Fig. 1a,b; ref. 7). This feeding diversity relies on the presence of moveable teeth and *Pristionchus* nematodes exhibit two distinct morphs—stenostomatous (St, narrow-mouthed) or eurystomatous (Eu, wide-mouthed) —that differ in the number and shape of associated teeth and the size and form of the buccal cavity^8^ (Fig. 1c,d). When fed on *Escherichia coli* OP50 bacteria under lab conditions, *P. pacificus* California reference strain RS2333 hermaphrodites have a stable 70:30% Eu:St ratio, but this can be influenced by starvation, crowding and pheromone signalling^8^,^9^,^10^. Because *P. pacificus* hermaphrodites reproduce primarily by selfing, strains are genetically homogeneous, and the presence of two distinct morphs thus represents an example of developmental plasticity, which was also demonstrated experimentally^8^.

The existence of developmental switch mechanisms is essential for the irreversible control of plasticity and has long been anticipated by evolutionary theory^1^, but associated mechanisms are largely unknown. We have recently identified the sulfatase *eud-1* as part of a genetic network that constitutes the developmental switch for the *P. pacificus* mouth-form decision^6^. In *eud-1* mutants, the Eu form is absent (*eud*, *eu*rystomatous-form-*d*efective), whereas overexpression from transgenes fixes the Eu form, thus confirming that EUD-1 acts as a developmental switch^6^. *eud-1* is X-linked and dosage-dependent, and it regulates differences in mouth-form frequency between hermaphrodites and males, among *P. pacificus* strains, and between *Pristionchus* species^6^. Interestingly, *P. pacificus eud-1* derives from a recent duplication that resulted in two neighbouring gene copies arranged in a head-to-head orientation (Fig. 1e). *eud-1* is expressed in a small number of *P. pacificus* head neurons, where its expression is sufficient to induce the execution of the Eu mouth-form^6^. However, while *eud-1* expression is highly regulated, the underlying mechanisms that control this developmental switch gene remain unknown.

Here we show that mutations in the conserved histone acetyltransferase *Ppa-lsy-12* and the methyl-binding-protein *Ppa-mbd-2* result in the absence of the Eu mouth-form similar to mutants in *Ppa-eud-1*. Mutations in both genes cause histone modification defects that result among others, in reduced *eud-1* expression. In addition, in *Ppa-lsy-12* mutants an antisense-*eud-1* RNA is also down-regulated. Overexpression of the antisense-*eud-1* RNA from transgenes increases the *eud-1*-sensitive mouth-form and results in increased *eud-1* expression. In contrast, this effect is absent in *eud-1* mutants indicating that antisense-*eud-1* positively regulates *eud-1*. These epigenetic mechanisms provide a theoretical framework for linking genetic regulation to environmental input.

## Results

### Two pleiotropic mutants with mouth-form defects

To study the regulation of *eud-1*, we searched for pleiotropic mutants with a Eud phenotype in hermaphrodites, similar to *eud-1*. By screening more than 30 already-established mutant strains with egg laying- or vulva defects, we identified two mutants, *tu319* and *tu365*, with a nearly complete loss of the Eu form (Fig. 2a). While *tu319* was previously molecularly uncharacterized, *tu365* represents a deletion allele in the methyl-binding protein family member *Ppa-mbd-2* (ref. 11). *Ppa-mbd-2(tu365)* is recessive, homozygous viable, and displays both a fully penetrant egg-laying defect and a complete absence of the Eu mouth-form (Fig. 2a). *Ppa-mbd-2(tu365)* contains a 1.7 kb deletion that removes four of six exons, suggesting that the absence of the Eu form results from a strong reduction-of-function or even null mutation in this gene. Thus, the phenotype of *mbd-2* in *P. pacificus* reveals the existence of pleiotropic regulators of mouth-form plasticity.

### A conserved histone-acetyltransferase regulates plasticity

In *tu319* mutants, only 2% of hermaphrodites have a Eu mouth-form (Fig. 2a). *tu319* was isolated in a screen for vulva-defective mutants and represents one of three alleles of the previously genetically characterized *vul-2* (*vul*valess) locus^12^. Interestingly, only *tu319* but not the two other alleles, *tu18* and *tu30*, show mouth-form defects indicating that the effect of *vul-2* on mouth-form regulation is allele-specific. We mapped *tu319* to the tip of chromosome IV in proximity to the marker S210 (Supplementary Fig. 1a). Sequencing of fosmid clones of this gene poor region resulted in the identification of a histone-acetyltransferase orthologous to the *Caenorhabditis elegans* gene *lsy-12* (Supplementary Fig. 1b,c; ref. 13. Sequencing of *lsy-12* identified mutations in all three alleles; *tu319* and *tu30* have splice-site mutations, whereas *tu18* contains a 598 bp deletion in the putative 3′-end of the gene (Supplementary Fig. 1b). *Ppa-lsy-12* is a complex gene with more than 30 predicted exons, and rapid amplification of cloned/cDNA ends (RACE) and RNA seq experiments provide strong evidence for extensive alternative splicing (Supplementary Fig. 1b). *Ppa-lsy-12* has a typical MYST domain in the 5′ part of the gene encoded by exons 5–13 (Supplementary Fig. 1b), which is present in the majority of transcripts. Interestingly, *tu319* affects the splice site of exon 7, whereas the two other alleles affect the 3′ part of the gene, which is not associated with known protein domains and is not present in the majority of transcripts.

To attempt phenotypic rescue, we generated a construct of *Ppa-lsy-12* containing exons 1–20 (see Methods) and obtained three independently transformed lines carrying the *Ppa-lsy-12* construct alongside an *egl-20::rfp* (red fluorescent protein) reporter. All three transgenic lines rescued both the vulvaless defect and the mouth-form defect of *tu319* (Supplementary Fig. 1c,d). Specifically, in transgenic animals the mouth-form was 71% Eu (versus 2% Eu in *tu319* worms) and, in one line studied in greater detail, 90% of the vulva precursor cells were induced to form vulval tissue (versus 33% in *tu319* worms). These results indicate that *vul-2* is indeed identical to *Ppa-lsy-12* and we renamed the gene accordingly. Taken together, two evolutionarily conserved genes, *Ppa-lsy-12* and *Ppa-mbd-2*, are pleiotropic regulators of mouth-form plasticity and mutations in these genes result in a strong reduction or absence of the Eu mouth-form.

### *mbd-2 and lsy-12* mutants cause histone modification defects

The molecular nature of *Ppa-lsy-12* suggests that chromatin remodelling may control the developmental switch mechanism that underlies the *P. pacificus* mouth dimorphism. Chromatin remodelling proteins regulate numerous developmental processes^14^, but nothing is known of a potential role for chromatin remodelling in the regulation of developmental plasticity. Therefore, we first asked if histone modifications are indeed altered in *lsy-12* and *mbd-2* mutants. We isolated proteins from mixed stage cultures of wild-type, *mbd-2*, and *lsy-12* mutant animals and found changes of four histone marks using antibody staining (Fig. 2b). H3K4me3 is strongly reduced in both *mbd-2* and *lsy-12* mutant animals, whereas H3K4me2 is reduced only in *mbd-2* mutants (Fig. 2b). In contrast, H3K4me1 and several other histone marks are not affected (Supplementary Fig. 2a). In addition to H3K4 methylation, the acetylation of H3K27 is strongly, and that of H3K9 moderately, reduced in both mutants (Fig. 2b). These findings demonstrate a genome-wide role for MBD-2 and LSY-12 in histone modifications in *P. pacificus.* Furthermore, because H3K4 methylation and acetylation of various H3 lysines are often found as gene activation marks^14^, these results suggest that the effects of *mbd-2* and *lsy-12* on mouth-form developmental plasticity is a consequence of chromatin remodelling-mediated transcriptional regulation.

### *eud-1* expression is down-regulated in *lsy-12* mutants

Next, we tested the developmental switch gene *eud-1* as a potential candidate target of chromatin remodelling by LSY-12 and MBD-2. First, we studied *eud-1* expression by performing quantitative reverse transcription (qRT)–PCR experiments in wild-type and mutant hermaphrodites in different developmental stages. Indeed, *eud-1* is significantly down-regulated in *mbd-2* and *lsy-12* mutants, in J2 worms, the larval stage at which the mouth-form is determined (Fig. 2c). In addition, we also observed *eud-1* down-regulation in adult stages, suggesting that *eud-1* expression is similarly controlled throughout development (Supplementary Fig. 2b). These results suggest that the mouth-form defects of *mbd-2* and *lsy-12* mutants result from down-regulation of *eud-1*. Interestingly, these effects of *mbd-2* and *lsy-12* mutants on *eud-1* expression levels and the mouth-form frequency qualitatively match the patterns seen in *P. pacificus* males and highly St wild isolates^6^. Altogether, our findings indicate that reduction-of-function or loss-of-function mutations in *mbd-2* and *lsy-12* cause genome-wide changes in histone modifications, which alter, among other target genes, the expression of *eud-1* throughout development.

### An antisense RNA associated with the *eud-1* locus

To further explore the function of chromatin remodelling on the regulatory network controlling the developmental switch, we used ultra-directional RNAseq to compare the transcriptomes of wild-type and *Ppa-lsy-12* mutant animals (Fig. 3a). In total, we found 309 genes to be differentially expressed (Supplementary Data 1). This includes, consistent with our qRT–PCR results *eud-1* expression, which was heavily down-regulated in *Ppa-lsy-12* worms (*P*<10^−8^, Fisher exact test). Surprisingly, however, we also found a strong effect on previously uncharacterized antisense reads at the *eud-1* locus (Fig. 3a). Indeed, additional RT–PCR experiments identified an antisense *eud-1* transcript, termed as-*eud-1*. The as-*eud-1* RNA consists of three exons with a total size of 863 nucleotides, some of which cover *eud-1* exons, such as exons 7–10 and exon 19 (Fig. 3a). When we searched for short open reading frames we did not observe any evidence for coding potentials and putative micropeptides longer than 10 amino acids. Thus, as-*eud-1* has no obvious open reading frame suggesting that as-*eud-1* encodes a long non-coding (lnc) RNA (Supplementary Fig. 3).

### *eud-1* and as-*eud-1* are co-expressed

Next, we tried to determine the expression pattern of as-*eud-1*. First, we used various constructs containing a putative 3.5 kb as-*eud-1* promoter fragment fused to turbo-RFP but they did not reveal specific expression. Therefore, we established RNA fluorescent *in-situ* hybridization (FISH) protocols. Indeed, we were able to detect *eud-1* RNA in the same head neurons as previously reported for a 7 kb *eud-1* promoter fragment driving RFP expression (Fig. 4a, Supplementary Fig. 4a; ref. 6). FISH of the as-*eud-1* RNA and *eud-1* RNA revealed that both transcripts are indeed expressed at the same site (Fig. 4b, Supplementary Fig. 4b; Supplementary Movie). Thus, our experiments suggest that *eud-1* and as-*eud-1* are co-expressed.

### Overexpression of as-*eud-1* increases the Eu mouth-form

**Figure 1:**
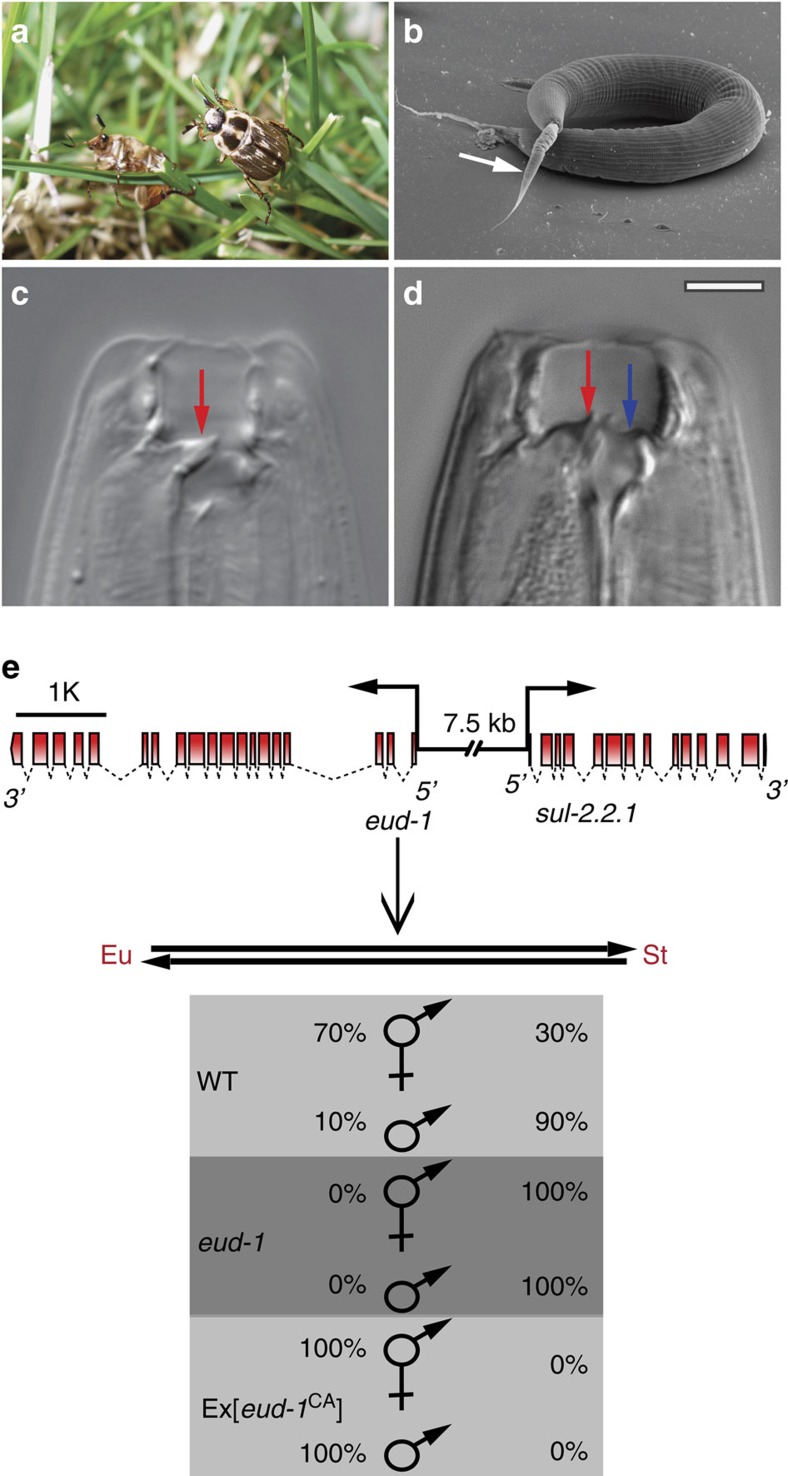
Developmental plasticity in *P. pacificus* and its regulation by the developmental switch gene *eud-1*. (**a**) The oriental beetle *Exomala orientalis* is one of the beetle hosts with which *P. pacificus* lives in a necromenic association. (**b**) Scanning electron micrograph showing *P. pacificus* predatory feeding on a small larva of *C. elegans* (white arrow). (**c**,**d**) Mouth dimorphism of *P. pacificus* enabling a switch between bacterial grazing and predatory feeding. Stenostomatous (St) animals (**c**) have a narrow buccal cavity and a flint-like dorsal tooth (red arrow), but miss the subventral tooth. In contrast, eurystomatous (Eu) animals (**d**) have a wide buccal cavity, a claw-like dorsal tooth (red arrow) and an additional subventral tooth (blue arrow). Scale bars, 10 μm. (**e**) Molecular organization of the *eud-1* locus and effect of *eud-1* function on mouth-form ratios. *eud-1* derives from a recent gene duplication, with the neighbouring sulfatase *sul-2.2.1* arranged in a head-to-head orientation. The two genes are separated by a 7.5 kb intergenic region that when used as promoter drives the expression of *eud-1* in various head neurons. In wild-type animals, hermaphrodites and males form ∼70% and 10% Eu animals, respectively. In *eud-1* mutants, both sexes are completely St, whereas *eud-1* overexpression causes both genders to form only Eu animals indicating that EUD-1 functions as developmental switch.

**Figure 2:**
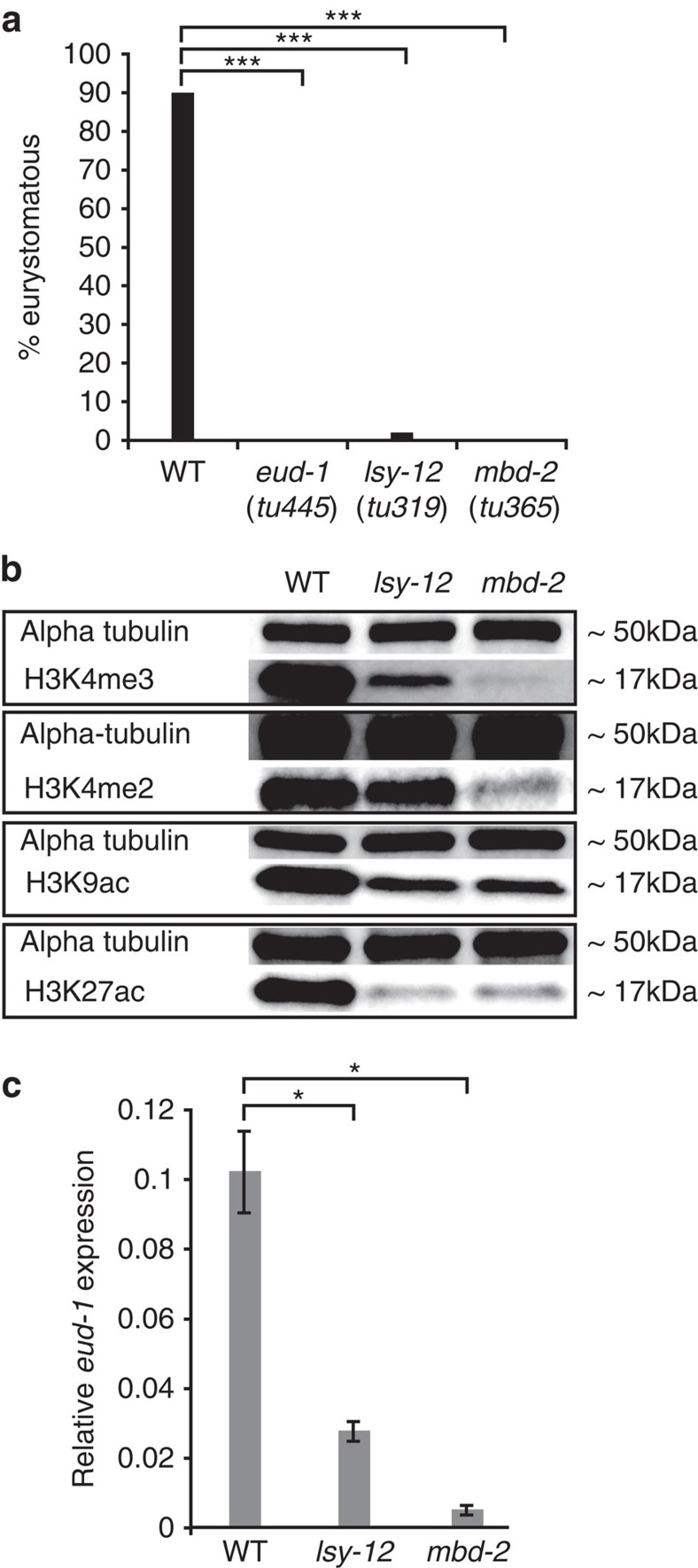
Mouth-form defects of two pleiotropic mutants and their effect on histone modification and *eud-1* expression. (**a**) *Ppa-lsy-12(tu319)* and *Ppa-mbd-2(tu365)* result in the (nearly) complete absence of Eu hermaphrodites, similar to *eud-1* mutants. Data are presented as the total Eu frequency, *n*>100 for all strains. (**b**) *Ppa-lsy-12* and *Ppa-mbd-2* mutants result in severe histone modification defects. This experiment has been replicated three times. (**c**) qRT–PCR experiments reveal down-regulation of *eud-1* expression in *Ppa-lsy-12* and *Ppa-mbd-2* mutants relative to wild type in J2 larvae. This experiment has been replicated three times. Error bars are defined as s.e.m. *P<0.05 and ***P<10^−5^, Kruskal–Wallis test.

To study the functional significance of this lnc RNA for the mouth-form decision, we performed overexpression experiments of as-*eud-1*. Specifically, we generated transgenic animals in which the as-*eud-1* complementary DNA (cDNA) is placed under the *eud-1* promoter, because the putative as-*eud-1* promoter region itself does not drive specific expression of the antisense transcript (see above). Given that *eud-1* and as-*eud-1* are co-expressed the use of the *eud-1* promoter presumably results in overexpression of as-*eud-1*. We generated transgenic animals in a wild-type background in order to be able to score the potential effects of as-*eud-1*. We obtained three independent transgenic lines, all of which showed a strong masculinization phenotype resulting in more than 95% of animals being males. These transgenic lines showed no embryonic lethality and transgenic males were successfully mated indicating that the high incidence of males result from as-*eud-1*-induced X chromosome-specific non-disjunction, a phenomenon known from various *C. elegans* mutants such as *him-8* (ref. 15). We, therefore, used the male mouth-form frequency to study the influence of as-*eud-1*. In contrast to hermaphrodites, spontaneous wild-type males are only 10–20% Eu because *eud-1* is X-linked and dosage-sensitive (Fig. 1e; refs 6, 10 ). The male mouth-form phenotype should be shifted towards more St animals in case of a negative effect and towards higher Eu frequencies in case of a positive function of the as-*eud-1*RNA.

**Figure 3:**
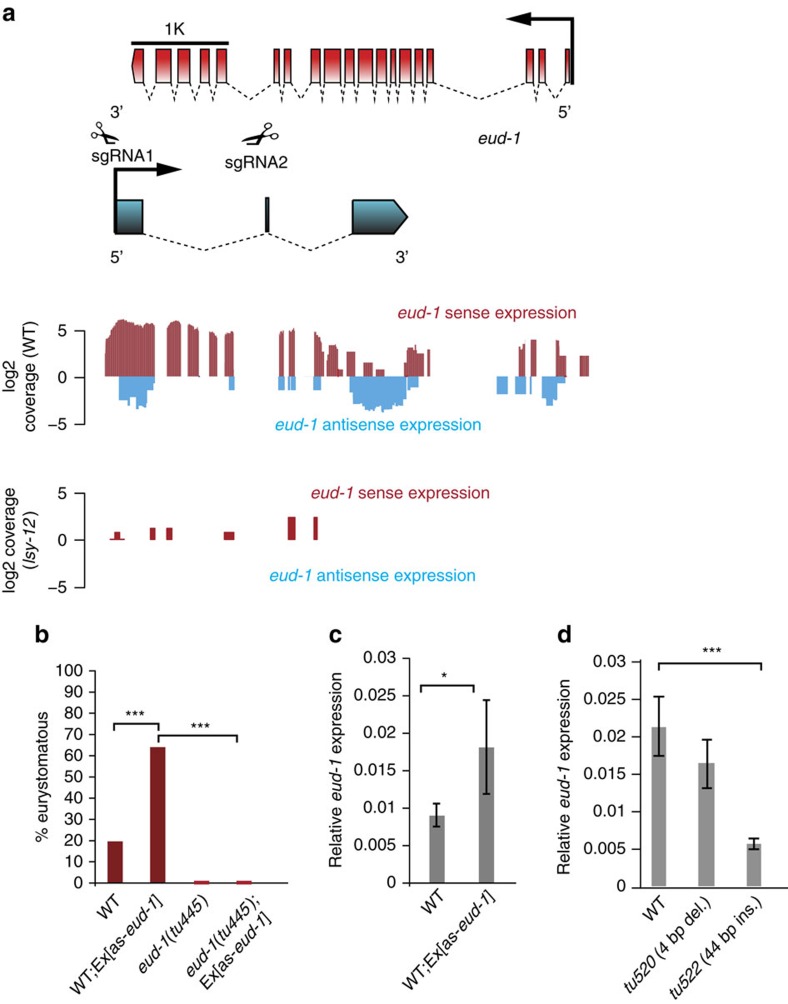
Molecular organization and function of as-*eud-1*. (**a**) Organization of the *eud-1* and antisense *eud-1* (as-*eud-1*) locus and RNAseq experiments of wild-type and *Ppa-lsy-12* mutant animals. The long noncoding RNA as-*eud-1* consists of three exons that span large parts of the *eud-1* coding region. The structure of as-*eud-1* was identified in RT–PCR experiments and revealed the existence of a short exon, which went undetected in RNAseq. Other antisense reads obtained at lower frequency in the RNAseq experiment, were not confirmed to be part of as-*eud-1* in RT–PCR experiments with mixed stage wild-type animals. Subsequent panels show sense and antisense expression as measured for wild-type (wt) and *Ppa-lsy-12* mutant animals. Note that nearly no reads of *eud-1* and as-*eud-1* were observed in *Ppa-lsy-12* mutants. sgRNA1 and sgRNA2 in the 5′ untranslated region (UTR) and exon 2 of as-*eud-1*, respectively, were used to induce mutations by CRISPR. (**b**) Transformation of wild-type hermaphrodites with as-*eud-1* cDNA induced a high incidence of males and a Eud phenotype in male progeny. In contrast, transformation of *eud-1(tu445)* mutant animals with as-*eud-1* did not result in a Eud phenotype, although the high incidence of males was similar to the transformation of wild-type animals. Two independent transgenic lines were generated each, *n*>100 for all strains. (**c**) qRT–PCR experiments reveal an up-regulation of *eud-1* in as-*eud-1* transgenic males relative to wild-type males. This experiment has been replicated three times. Error bars are defined as s.e.m. (**d**) qRT–PCR experiments reveal that *eud-1* is significantly down-regulated in the as-*eud-1* promoter mutant *tu522* that contains a 44 bp insertion. This experiment has been replicated three times. Error bars are defined as s.e.m. *P<0.05 and ***P<10^−5^, Kruskal–Wallis test.

**Figure 4:**
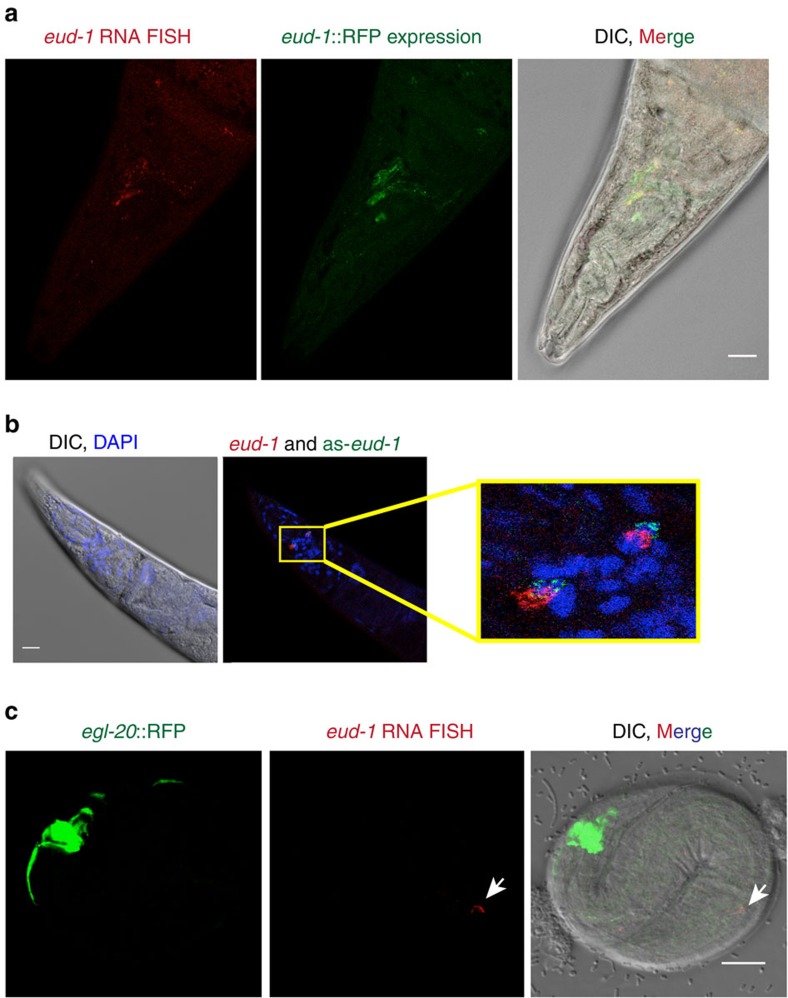
Fluorescent *in situ* hybridization (FISH) of *eud-1* and as-*eud-1* reveals co-expression of both transcripts. FISH probes were designed as described in the Methods section. Photographs in **a** and **b** show adult animals, photographs in **c** show a J1 stage larvae, which in *P. pacificus* is still in the egg shell. (**a**) *eud-1* FISH (red, left image) and an *eud-1*::RFP reporter construct (green, central image) show the same expression pattern in several head neurons. The image at the right represents a merger of both and differential interference contrast (DIC) microscopy. Note that not all *eud-1*-expressing cells are visible in this plane of focus. (**b**) Head area of an adult worm with DIC and 4,6-diamidino-2-phenylindole staining (left image) and co-expression of *eud-1* (red) and as-*eud-1* (green) as revealed by FISH probes. Both transcripts are expressed at multiple foci, two of which are shown in this plane of focus (inset). Overlapping fluorescence (yellow) was seen in all animals observed, but not in all cells. The expression pattern was highly consistent among multiple adults (*n*>20). See Supplementary Movie for additional details of the partially overlapping expression of both transcripts. (**c**) Transgenic animals carrying an *eud-1*::as-*eud-1* construct show *eud-1* expression in head neurons already in the J1 stage, which is never seen in wild-type animals. *egl-20*::RFP (green, left image) is used as transformation marker. The same *eud-1* FISH probe (red, central image) was used as above. The image at the right represents a merger and DIC microscopy. Supplementary Fig. 4 provides additional photographs for **a** and **b**. Scale bars, 10 μm.

We made the remarkable finding that as-*eud-1* has a positive function on the Eu versus St mouth-form decision and *eud-1* expression (Fig. 3b,c), whereas most cases of antisense-mediated regulation results in transcriptional down-regulation^16^. Four observations result in this conclusion. First, the frequency of the *eud-1*-sensitive mouth-form was dramatically increased in transgenic lines carrying as-*eud-1* in a wild-type background (from 20 to 64% Eu males) (Fig. 3b). Second, qRT–PCR experiments revealed a strong up-regulation of *eud-1* in as-*eud-1* transgenic males (Fig. 3c). Third, *eud-1* RNA can be detected in the J1 stage in *eud-1*::as-*eud-1* transgenic animals, an early expression that is never seen in wild-type animals (Fig. 4c). Finally, as-*eud-1* transgenes in a *eud-1* mutant background also caused a high incidence of males, but without affecting male mouth-form. Specifically, *eud-1(tu445)*;Ex(as-*eud-1*) males were completely St (Fig. 3b), indicating that as-*eud-1* acts through *eud-1*. Taken together, these experiments suggest that chromatin remodelling acts through antisense-mediated up-regulation of *eud-1*.

Finally, we used the recently developed CRISPR/Cas9 technology in *P. pacificus* (ref. 17) to generate mutations that would specifically affect as-*eud-1*, but not *eud-1*. Therefore, we targeted the small exon 2 of as-*eud-1*, but were unable to generate a deletion/insertion in this 26 bp exon (Fig. 3a). In contrast, we succeeded in generating two mutations in the putative promoter of as-*eud-1* (Fig. 3a). Specifically, *tu520* eliminates a 4 bp fragment, whereas *tu522* contains a 44 bp insertion. Both mutant lines show a wild-type mouth-form ratio. However, the *tu522* mutant shows significantly reduced *eud-1* expression as observed by qRT–PCR experiments (Fig. 3d). In contrast, qRT–PCR experiments with as-*eud-1* failed to reveal transcripts above background level, a phenomenon known from other lnc RNAs^18^. Altogether, these experiments provide further evidence that as-*eud-1* up-regulates *eud-1* expression and additionally, they suggest that as-*eud-1* expression is itself driven by distal regulatory elements that are unaffected in the *tu520* and *tu522* mutants.

## Discussion

Developmental switching represents an appealing concept to link genetic and environmental influences on phenotypically plastic traits. Our studies of the sulfatase *eud-1* —its function as a developmental switch, its role in micro- and macroevolutionary divergence and, here its regulation—provide such mechanistic insights. Previous characterization of *eud-1* resulted in several surprising findings, that is its recent origin by gene duplication and its epistasis to other factors controlling feeding plasticity^6^. We have now shown that two evolutionarily conserved genes, *mbd-2* and *lsy-12*, are involved in genome-wide histone modifications that also influence transcription of *eud-1*, providing first insight into the molecular mechanisms underlying the regulation of developmental switches. In particular, the involvement of antisense-mediated up-regulation of *eud-1* indicates an unexpected complexity and results in three major conclusions. First, our findings demonstrate that the role of *eud-1* involves complex regulation of its own transcription. We previously observed that the coding region of *eud-1* is subject to strong purifying selection, and our new findings support and extend these conclusions regarding the importance of regulatory versus structural changes. Second, we demonstrate the involvement of chromatin remodelling in the developmental switch mechanism regulating mouth-form plasticity in *P. pacificus*. We speculate that chromatin remodelling represents a powerful epigenetic mechanism that might link environmental signals to transcriptional regulation of plasticity. Third, we provide evidence for an antisense RNA in up-regulation. Transcriptional surveys of many eukaryotes have uncovered hundreds of noncoding transcripts^19^ and though many of these function as transcriptional regulators, most do so as inhibitors. Conversely, antisense-mediated transcriptional activation or maintenance has only rarely been described^18^,^20^. Thus, the example of as-*eud-1* regulation of *eud-1* reveals complex regulatory mechanisms that can serve as model to link genetic and environmental control of developmental plasticity in future studies.

## Methods

### Culture conditions

All wild-type worms were *P. pacificus* reference strain RS2333. All *Pristionchus* strains were kept on 6-cm plates with nematode growth medium agar and were fed with a lawn of *E. coli* OP50 grown in 400 μl L-broth. Cultures were maintained at 20 °C. Because the mouth-form ratio is sensitive to unknown environmental factors^6^, all experiments include their own controls for the wild-type Eu frequency. Also, to minimize the potential for laboratory evolution of the trait, a new culture of the California (RS2333) strain was revived annually from a frozen voucher.

### Phenotype scoring

The mouth-form phenotype was scored using the following characters to discriminate between Eu and St individuals, respectively, (i) the presence versus absence of a subventral tooth, (ii) a claw-like versus flint-like or triangular dorsal tooth, and (iii) a wide versus narrow stoma (mouth). Characters (i) and (ii) were discrete, non-overlapping, and sufficient to distinguish the two forms. Apparent intermediates between the two forms were rare (<0.1%) and were not included in counts. Phenotypes could be scored using Zeiss Discovery V.12 stereomicroscopes and were supplemented where necessary with differential interference contrast (DIC) microscopy on a Zeiss Axioskop.

### Mapping of *vul-2(tu319)* and mutant identification

For genetic mapping, mutants in the California background were crossed to the Washington strain (PS1843). F2 progeny were cloned and screened for two generations to confirm the mutant phenotype and the homozygosity of mutations. Genomic DNA of outcrossed mutant lines was extracted for genetic mapping. Simple-sequence conformation polymorphism markers were tested against 30–40 outcrossed mutant lines and detected as previously described^21^,^22^. *vul-2* was mapped to the tip of chromosome IV close to the marker S210. Further mapping localized *vul-2* to the bacterial-artificial-chromosome clone BACPP16-M16 and the fosmid subclones 525-J06, 543-P16 and 558-O23. Light shotgun sequencing of these fosmid clones resulted in the identification of *Ppa-lsy-12* as candidate gene for *vul-2*. To prepare samples for whole-genomic sequencing, DNA was extracted from all three alleles *tu18*, *tu30* and *tu319* and mutations were identified in all three alleles.

### Alternative splicing of *Ppa-lsy-12*

Following preparation of mixed-stage RNA libraries for *P. pacificus* RS2333, coding DNA (cDNA) was amplified by reverse transcription PCR and sequenced. 5′ and 3′ RACE experiments were performed by SMARTer RACE cDNA Amplification Kit following the manufacturer's protocol (Life Technologies). The full list of gene-specific primers that were designed according to the available genomic sequence for *Ppa-lsy-12* is provided in Supplementary Table 1.

### RNA-sequencing experiments

The presence and levels of gene expression were measured by whole-transcriptome sequencing (RNA-Seq) of *Ppa-lsy-12(tu319)* mutants and *P. pacificus* wild type. Culture populations were allowed to grow until their food was exhausted, immediately after which the cultures were processed for sequencing. Five mixed-stage plates were washed with 40 ml M9, centrifuged immediately at 1300 *g* for 4 min, rinsed with 40 ml 0.9% NaCl treated with 40 μl ampicillin and 40 μl chloramphenicol and shaken gently for 2 h, and finally concentrated into a pellet by centrifugation and immediately frozen in liquid nitrogen. NEBNext Ultradirectional RNA Library Kit (Cat # E7420L) was used to prepare libraries. RNA-Seq libraries were sequenced as 2 × 100-bp paired-end reads on an Illumina HiSeq 2000, yielding 30.6 million reads for the wild type and 31.6 million reads for the *lsy-12* sample. Raw reads were aligned to the reference genomes of *P. pacificus* (Hybrid1) (www.pristionchus.org), using the software Tophat v.2.0.3 (ref. 23). Expression levels were estimated and tested for differential expression using the programs Cufflinks and Cuffdiff v.2.0.1 (ref. 23) resulting in 95 significantly differentially expressed genes including *eud-1* (FDR corrected *P* value<0.01). The equivalent test for down-regulation of as-*eud-1* was not significant despite no evidence of as-*eud-1* expression in *Ppa-lsy-12* animals, which is probably due to the reduced statistical power for differential expression detection resulting from the low expression of as-*eud-1* even in wild-type animals.

### qRT–PCR

Total RNA from synchronized cultures was isolated using TRIzol (ambion by life technologies). For reverse transcription Superscript II reverse transcriptase (Invitrogen, Cat. No: 18064) was used following the manufacturer's instructions. We used 1 μg total RNA. The qRT–PCR experiments were performed on a LightCycler 480 system; using SybrGreen (Roche Diagnostics) with a reaction set up described elsewhere^24^. To detect *eud-1* expression we used VSe13F GATGATCGAGTCACACAGATCCG forward and VSe13R ATGTAGTAGGAGAGTTGAGCAGCG reverse primers. *Ppa-cdc-42* and *Ppa-Y45F10D.4* were used as reference genes as previously described^25^. PCR efficiencies were determined using external standards on plasmid mini-preparation of cloned PCR-products. Expression levels were analysed by basic relative quantification. We performed 3–6 biological replicates for different experiments.

### as-*eud-1* transcript analysis

RNAseq reads of wild-type worms cover the majority of *eud-1* exons to a similar extent. In addition, we observed antisense readsat the *eud-1* locus that were previously uncharacterized. These antisense reads are expressed at very low levels and cannot be detected in qRT–PCR experiments, which otherwise are used as a standard procedure in *P. pacificus* (see above). We used many different PCR primer combinations (Supplementary Table 2) in a variety of nested PCR experiments to study which of the antisense reads if any are present in a potential as-*eud-1* cDNA. These experiments revealed the existence of one transcript of 863 nucleotides that consists of three exons (Fig. 3, Supplementary Fig. 5). The two larger exons cover exactly those reads that were most abundantly found in the RNAseq experiment of wild-type worms. However, exon 2 consists of only 26 nucleotides and went unnoticed at the RNAseq level. Similar to noncoding (nc) RNA (ncRNA) in other systems^18^, as-*eud-1* is expressed at very low levels.

### Genetic transformation

For phenotypic rescue of *vul-2*, the germ line of *vul-2(tu319)* mutant animals was injected with a 17 kb genomic construct containing exons 1-20 of *Ppa-lsy-12* and 4.5 kb of flanking regulatory region (2 ng ul^−1^), the marker *Ppa-egl-20*::*TurboRFP* (10 ng ul^−1^) and genomic carrier DNA (60 ng ul^−1^ from the recipient strain^26^. To study the as-*eud-1* lnc RNA, we generated a 7.5 kb construct consisting of ∼6.5 kb promoter element and the 860 bp cDNA fragment of as-*eud-1*. This construct was injected (2 ng ul^−1^) with the *Ppa-egl-20*::*TurboRFP* (10 ng ul^−1^) marker and genomic carrier DNA (60 ng ul^−1^) of *P. pacificus* RS2333 and *Ppa-eud-1(tu445)*, respectively. For all constructs, at least two independent transgenic lines were generated and transgenic animals were scored over multiple generations involving at least 100 transgenic animals per line. Transgenic lines containing the as-*eud-1* lnc RNA yielded more than 90% male progeny and all lines were kept at least until the tenth generation. No embryonic lethality was observed in association with these transgenes. Transgenic males were crossed with *Ppa-pdl-1* and wild-type hermaphrodites and cross-progeny was readily observed.

### CRISPR/Cas9 induced mutations

To generate CRISPR/Cas9 induced mutations, sgRNAs were co-injected with Cas9 protein^17^. We used the following sites for single-guided (sg) RNAs (sgRNA):

sgRNA1: 5′ CAGTTGAAGAACAAAACACACGG 3′.

sgRNA2: 5′ GTCGTAATCAAGCTAACAGCTGG 3′.

### Statistical analyses

All phenotypic data show Eu frequency calculated from total individuals screened. Total sample size is illustrated on graphs. Significant differences were tested by Fisher's exact test. For the expression data we performed Kruskal–Wallis test. All statistical analyses were implemented in the program Statistica v. 9 (Statsoft).

### Western blotting and antibodies

Proteins were extracted from mixed stage cultures. Concentration was determined by Neuhoff's Dot-Blot assay^24^. Proteins were equally loaded and separated in polyacrylamid gels. Proteins were transferred to polyvinylidene difluoride transfer membrane and incubated overnight with primary antibodies (Supplementary Table 3), and were then incubated for an hour in secondary antibodies (Anti-rabbit IgG, horseradish peroxidase-linked antibody, Cell Signaling Technology, Cat. #7074S and Anti-mouse IgG, horseradish peroxidase-linked antibody, Cell Signaling Technology, Cat. #7076S). For dilution of primary antibodies see Supplementary Table 3. The secondary antibody was diluted 1:1,000. The detection was done by Bio-Rad Clarity western enhanced chemiluminescent (ECL) substrate using Peqlab FUSION Xpress multi-imaging system. We provide an uncropped scan of the most important blot as Supplementary Fig. 5.

### Single molecule RNA FISH

Single molecule RNA FISH was performed using a protocol described earlier for *C. elegans*^25^. Biosearch Technologies Stellaris FISH online platform was used to design and order *eud-s* and *as-eud-1* probes. They were coupled with Quasar 670 and TAMRA fluorescent dyes, respectively. Image acquisition was performed on Leica SP8 confocal system using settings to maximize detection of fluorescent dyes. Image J software was used for Image analysis.

### Data availability

All relevant data, including mutant and transgenic lines, constructs and plasmids are available upon request from the corresponding author^26^.

## Additional information

Accession Codes: The RNA-seq data are available at the NCBI sequence read archive under accession codes SRX1609204, SRX1858662, SRX1858663.

**How to cite this article:** Serobyan, V. *et al*. Chromatin remodelling and antisense-mediated up-regulation of the developmental switch gene *eud-1* control predatory feeding plasticity. *Nat. Commun.* 7:12337 doi: 10.1038/ncomms12337 (2016).

## Supplementary Material

Supplementary Information Supplementary Figures 1-5, Supplementary Tables 1-4

Supplementary Movie 1 Fluorescent *in situ* hybridization indicating partial overlap of the *eud-1* and as-*eud-1* transcripts.

Supplementary Data 1 Differentially expressed genes between wild type and Ppa-lsy-12 mutant animals (blue, up-regulated; green, down regulated).

## References

[b1] West-Eberhard M.-J. Developmental Plasticity and Evolution Oxford University Press (2003).

[b2] Moczek A. P. . The role of developmental plasticity in evolutionary innovation. Proc. R. Soc. B 278, 2705–2713 (2011). 10.1098/rspb.2011.0971 PMC3145196 21676977

[b3] Nijhout H. F. Insect Hormones Princeton University Press (2003).

[b4] Schlichting C. D. Origins of differentiation via phenotypic plasticity. Evol. Dev. 5, 98–105 (2003). 12492416 10.1046/j.1525-142x.2003.03015.x

[b5] Pigliucci M. Phenotypic Plasticity: Beyond Nature and Nurture John Hopkins University (2001).

[b6] Ragsdale E., Müller M., Roedelsperger C. & Sommer R. J. A developmental switch coupled to the evolution of plasticity acts through a sulfatase. Cell 155, 922–933 (2013). 24209628 10.1016/j.cell.2013.09.054

[b7] Herrmann M. . The nematode *Pristionchus pacificus* (Nematoda: Diplogastridae) is associated with the Oriental beetle *Exomala orientalis* (Coleoptera: Scarabaeidae) in Japan. Zool. Sci. 24, 883–889 (2007). 17960992 10.2108/zsj.24.883

[b8] Bento G., Ogawa A. & Sommer R. J. Co-option of the endocrine signaling module Dafachronic Acid-DAF-12 in nematode evolution. Nature 466, 494–497 (2010). 20592728 10.1038/nature09164

[b9] Bose N. . Complex small molecular architectures regulate phenotypic plasticity in a nematode. Angew. Chemie. 51, 12438–12443 (2012). 10.1002/anie.201206797 PMC3733369 23161728

[b10] Serobyan V., Ragsdale E., Müller M. & Sommer R. J. Feeding plasticity in the nematode *Pristionchus pacificus* is influenced by gender and social context and is linked to developmental speed. Evol. Dev. 15, 173–182 (2013). 10.1111/ede.12030 23607300

[b11] Gutierrez A. & Sommer R. J. Functional diversification of the *mbd-2* gene between *Pristionchus pacificus* and *Caenorhabditis elegans*. BMC. Genet. 8, 57 (2007). 17725827 10.1186/1471-2156-8-57 PMC2000911

[b12] Sigrist C. & Sommer R. J. Vulva formation in *Pristionchus pacificus* relies on continuous gonadal induction. Dev. Genes Evol 209, 451–459 (1999). 10415322 10.1007/s004270050278

[b13] Sarin S. . Analysis of multiple ethyl methanesulfonate-mutagenized *Caenorhabditis elegans* strains by whole-genome sequencing. Genetics 185, 417–430 (2010). 20439776 10.1534/genetics.110.116319 PMC2881126

[b14] Musselman C. A., Lalonde M.-E., Cote J. & Kutateladze T. G. Perceiving the epigenetic landscape through histone readers. Nat. Struct. Mol. Biol. 19, 1218–1227 (2012). 23211769 10.1038/nsmb.2436 PMC3645987

[b15] Hodgkin J. A., Horvitz H. R. & Brenner S. Nondisjunction mutants on the nematode *Caenorhabditis elegans*. Genetics 91, 67–94 (1979). 17248881 10.1093/genetics/91.1.67 PMC1213932

[b16] Rinn J. L. & Chang H. Y. Genome regulation by long noncoding RNAs. Annu. Rev. Biochem. 81, 145–166 (2012). 22663078 10.1146/annurev-biochem-051410-092902 PMC3858397

[b17] Witte H. . Gene inactivation using the CRISPR/Cas9 system in the nematode *Pristionchus pacificus*. Dev. Genes Evol. 225, 55–62 (2015). 25548084 10.1007/s00427-014-0486-8

[b18] Dimitrova N. . *LincRNA-p21* activates *p21* in *cis* to promote polycompb target gene expression and to enforce the G1/S checkpoint. Mol. Cell 54, 1–14 (2014). 24857549 10.1016/j.molcel.2014.04.025 PMC4103188

[b19] Guttman M. . Chromatin signature reveals over a thousand highly conserved large non-coding RNAs in mammals. Nature 458, 223–227 (2009). 19182780 10.1038/nature07672 PMC2754849

[b20] Sakurai I. . Positive regulation of *psbA* gene expression by cis-encoded antisense RNAs in *Synechocystis* sp. PCC6803. Plant. Physiol. 160, 1000–1010 (2012). 22858634 10.1104/pp.112.202127 PMC3461525

[b21] Srinivasan J. . A bacterial artificial chromosome-based genetic linkage map of the nematode *Pristionchus pacificus*. Genetics 162, 129–134 (2002). 12242228 10.1093/genetics/162.1.129 PMC1462235

[b22] Srinivasan J. . An integrated physical and genetic map of the nematode *Pristionchus pacificus*. Mol. Genet. Genomics 269, 715–722 (2003). 12884007 10.1007/s00438-003-0881-8

[b23] Trapnell C. . Differential gene and transcript expression analysis of RNA-Seq experiments with TopHat and Cufflinks. Nat. Protoc. 7, 562–578 (2012). 22383036 10.1038/nprot.2012.016 PMC3334321

[b24] Neuhoff V., Philipp K., Zimmer H.-G. & Mesecke S. A simple, versatile, sensitive and volume-independent method for quantitative protein determination which is independent of other external influences. Hoppe-Seyler's Z. Physiol. Chem. 360, 1657–1670 (1979). 92445 10.1515/bchm2.1979.360.2.1657

[b25] Ji N. & van Oudenaarden A. Single Molecule Fluorescent In Situ Hybridization (smFISH) of C. elegans Worms and Embryos The *C. elegans* Research Community. Wormbook, ed. ((2012). 10.1895/wormbook.1.153.1 PMC4781154 23242966

[b26] Schlager B., Wang X., Braach G. & Sommer R. J. Molecular cloning of a dominant Roller mutant and establishment of DNA-mediated transformation in the nematode model *Pristionchus pacificus*. Genesis 47, 300–304 (2009). 19298013 10.1002/dvg.20499

