# Supplementary material for: Chromatin remodelling and antisense-mediated up-regulation of the developmental switch gene eud-1 control predatory feeding plasticity
Source: Nat Commun. 2016 Aug 4;7:12337. doi: 10.1038/ncomms12337 (PMC4976200; doi:10.1038/ncomms12337)
Supplement: Supplementary Information — Supplementary Figures 1-5, Supplementary Tables 1-4 [file ncomms12337-s1.pdf]

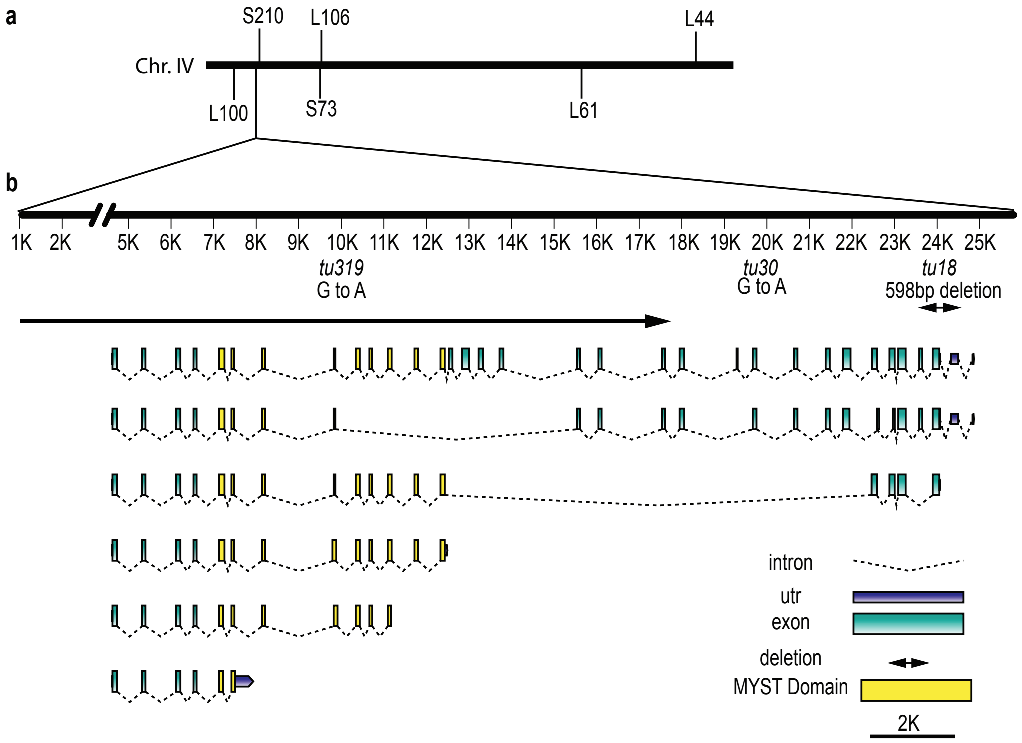

c

	WT	*lsy-12 (tu319)*	*tu319* Ex[*lsy-12*]
induction index	3	0.97	2.7
number of animals	many	72	10

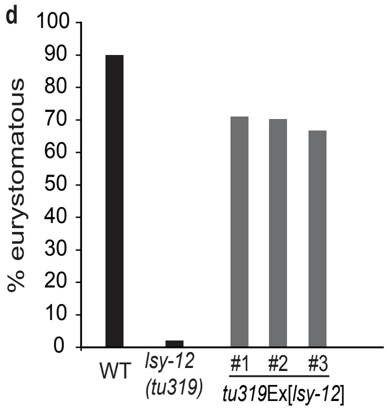

Supplementary Figure 1: Mapping, gene structure and rescue of *P. pacificus lsy-12*. (**a**) Genetic map showing the tip region of chromosome IV. (**b**) Exon – intron structure of *Ppa-lsy-12* deduced from cDNA cloning and sequencing and molecular nature of mutations. The various shown isoforms

are ordered by length. Note, that this list represents just a subset of the observed isoforms. Like in *C. elegans*, multiple isoforms exist and more than 30 exons are predicted for the *Ppa-lsy-12* gene. The successful rescue experiment indicated below were carried with a genomic fragment of approximately 17 kb covering 20 predicted exons as indicated by the arrow above the isoforms. (**c**) Rescue experiment of the vulva defect of *tu319*. In wild type hermaphrodites, three vulval precursor cells are induced to form vulval tissue as indicated by an induction index of 3. *tu319* mutant animals have an average induction of 0.97 cells that is rescued to an average induction of 2.7 cells by an array carrying multiple copies of *Ppa-lsy-12*. Only one transgenic line was tested for the rescue of the vulval defect. (**d**) Rescue experiment of the mouth-form defect of *tu319*. *tu319* animals are 2% Eu, whereas wild type hermaphrodites are 90% Eu under the same cultural conditions. Three independent transgenic lines (#1-#3) carrying multiple copies of *Ppa-lsy-12*, show 60-70% Eu animals as indicated by the analysis of more than 100 animals. Data are represented as the total Eu frequency.

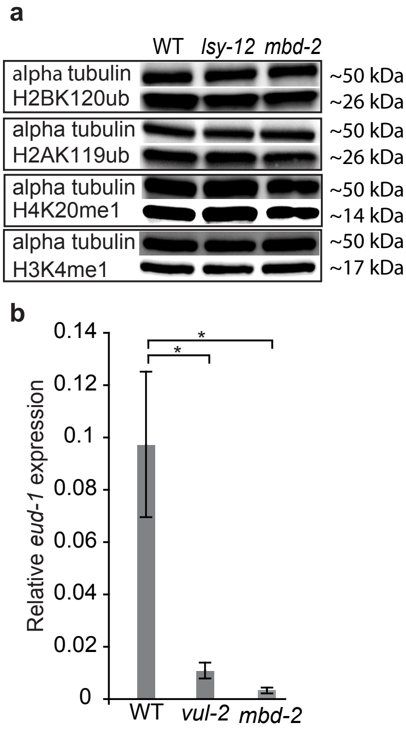

Supplementary Figure 2: Histone modification of additional histone markers and *eud-1* expression in adult animals. (**a**) *Ppa-lsy-12* and *Ppa-mbd-2* mutants do not result in obvious histone modification defects in several other tested histone marks. Here we show four representatives for ubiquitination (ub) and mono-methylation (me1). (**b**) qRT-PCR experiments reveal down regulation of *eud-1* expression in *Ppa-lsy-12* and *Ppa-mbd-2* mutants relative to wild type in adult worms. Error bars are s.e.m. *$p<0.05$ and ***$p<10^{-5}$, Kruskal-Wallis test.

ggaagacttccgcaagaagcatgcaaacactacattctaaatgatacaagagatttaacaatttactataataaatgtccagtt
gaagaacaaaacacacggagaaggttgtgaagggtttgcgaaagaactgatggaagcgagataaatcgttaggtatattta
ttcttattcaagctgatcgtattcttgatcactccagttgagttcttccttctcgagcatggctcttcgaagcgcgcgctggaatc
gatcactgcgtggagatgtagtagggtcgtaaaacaacgctaggtgggaccagggccgagtgggctacaagggaatcat
tatttgggattttaatactagcaaattagtacctcggcttttctctggtgaatgaagaacttaaaatcatcaaccaaattctgagtc
atatcgtcgaatatcatgggttgttgcaccaactcatcaccgtcatacaagaagcacttggttccattgggaccatcagggaa
cattgtctaaacaatctcgaaaattggtgttggtagatttttcagtaccttagtggtatcgcactcccatacatttgtgaagggaa
ggttgactcccacgtattgaaaaccacgacgggaaggcaaatgtactccgtcagtactggttctgaagcataaccatttaaa
gtgctgataattacaatttactaactcttcattgattccgagatgccatttgccgatcattcctgttgcgtaacctgaatttgcaatt
atttagatgtattcaatttcaattcccacctcgttcttcgaacatctcggccattgtagtctcatctttcggtagtcctccaatgtcct
gaggaacgaacactcgag

Supplementary Figure 3: Nucleotide sequence of the as-*eud-1* transcript as obtained in RT-PCR experiments.

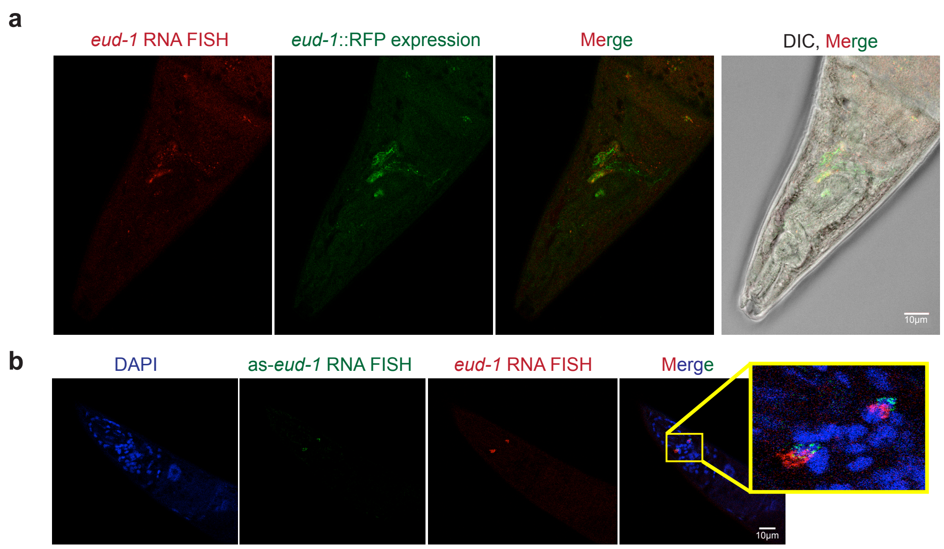

Supplementary Figure 4: Fluorescent *in situ* hybridization (FISH) of *eud-1* and as-*eud-1*. Photographs show adult animals and provide the same and additional photographs to those shown in Figure 4. (**a**) from left to right: i) *eud-1* FISH (red); ii) *eud-1*::RFP reporter construct (green); iii) merger of *eud-1* RNA FISH and *eud-1*::RFP expression showing overlap in yellow; iv) merger of both expressions with differential interference contrast (DIC) microscopy. (**b**) from left to right: i) DAPI staining; ii) as-*eud-1* RNA FISH (green); iii) as-*eud-1* (red); iv) co-expression of *eud-1* (red), as-*eud-1* (green) and DAPI.

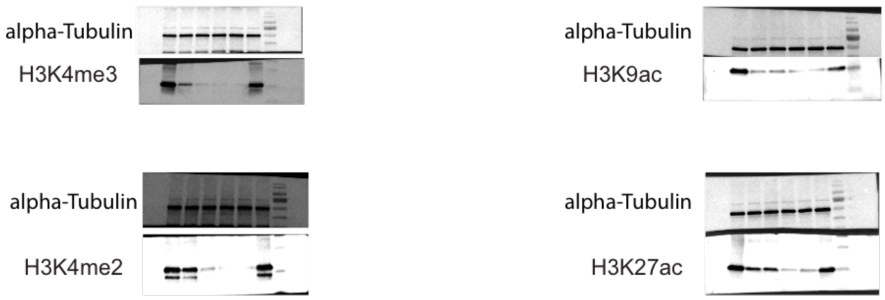

Supplementary Figure 5: Histone modification of *Ppa-lsy-12* and *Ppa-mbd-2* mutants result in severe histone modification defects. Un-cropped scans of blots provided in Figure 2b. Note that the original gels and blots contain additional lanes unrelated to this study. In each graph, the lanes relevant to this study are lanes 1-3 from the left, presenting wild type, *lsy-12* and *mbd-2* as shown in Figure 2b.

Supplementary Table 1: Differentially expressed genes between wild type and *Ppa-lsy-12* mutant animals (blue, up-regulated; green, down regulated).

P. pacificus Gene ID (version Hybrid1)	Expression foldchange	P (FDR corrected)
Contig41-snap.23	13,29000737	1,84319E-07
Contig22-snap.173	9,101661929	9,63592E-05
Contig11-snap.172	6,027375725	0,000110313
Contig70-snap.1	12,39485939	0,000128043
Contig12-snap.157	3,697320223	0,000149273
Contig106-snap.25	4,775295463	0,000300751
Contig10-snap.353	11,18109827	0,000317931
Contig41-snap.82	7,096117999	0,000919555
Contig77-snap.8	65,96916778	0,00100054
Contig143-snap.16	16,83556227	0,00105018
Contig4-snap.53	37,7475211	0,00123591
Contig22-snap.106	3,599433838	0,00179514
Contig52-snap.11	4,42065455	0,00284796
Contig29-snap.5	61,43291773	0,00333557
Contig30-snap.191	11,6756422	0,00333557
Contig65-snap.25	inf	0,00409407
Contig80-snap.15	3,577619607	0,0046836
Contig81-snap.24	14,3205004	0,00530466
Contig11-snap.371	2,536642133	0,00549032
Contig60-snap.86	87,23891972	0,00709599
Contig43-snap.76	5,296245508	0,00714069
Contig41-snap.5	4,873269472	0,00780334
Contig70-snap.59	3,294820797	0,00829626
Contig5-snap.322	79,30225582	0,00843321
Contig14-snap.271	3,385760678	0,00917801
Contig50-snap.180	2,637900229	0,00969665
Contig18-snap.207	78,60504474	0,0103225
Contig21-snap.203	5,691977662	0,0107445
Contig114-snap.47	3,607451503	0,01173
Contig70-snap.44	21,23570353	0,011961
Contig5-snap.109	73,6753447	0,0122673
Contig15-snap.100	26,78809343	0,012506
Contig2-snap.143	39,36795862	0,0133108
Contig43-snap.100	2,800452581	0,0143238
Contig122-snap.33	12,62892558	0,0143824
Contig11-snap.54	8,836162803	0,0170202
Contig45-snap.65	10,71500041	0,0205952
Contig0-snap.107	4,994949181	0,0206185
Contig45-snap.142	inf	0,0206552
Contig1-snap.244	4,337248188	0,0221536
Contig45-snap.89	12,50765998	0,0224134
Contig125-snap.20	3,921566323	0,0224164
Contig13-snap.199	2,791498924	0,0228273
Contig165-snap.14	inf	0,0268869
Contig91-snap.44	3,577669204	0,0272269
Contig13-snap.239	7,533132996	0,0280861
Contig36-snap.166	2,722458392	0,0282879
Contig93-snap.38	inf	0,0301426

Contig65-snap.26	8,275520184	0,0314173
Contig23-snap.131	9,258317253	0,0328285
Contig7-snap.59	3,557761992	0,0328329
Contig49-snap.90	3,471285437	0,0335256
Contig0-snap.498	2,38574525	0,03497
Contig5-snap.298	2,253129132	0,0364589
Contig29-snap.28	4,126704113	0,0379509
Contig12-snap.10	inf	0,0391579
Contig99-snap.10	2,124995815	0,039204
Contig155-snap.3	47,83915494	0,0394386
Contig12-snap.416	3,1659753	0,0409838
Contig3-snap.167	2,16899992	0,0409838
Contig125-snap.78	2,846699088	0,0411583
Contig11-snap.380	2,20643921	0,0434612
Contig43-snap.120	2,218062472	0,0438765
Contig78-snap.48	inf	0,0451409
Contig23-snap.197	54,92039132	0,0451409
Contig111-snap.8	45,22943284	0,0451409
Contig106-snap.1	18,20698332	0,0487759
Contig0-snap.648	3,022369399	0,0490452
Contig116-snap.30	3,456471428	0,0491213
Contig1-snap.120	inf	0,0493838
Contig28-snap.265	0,129880793	0
Contig97-snap.101	0,076369479	1,7466E-12
Contig56-snap.93	0,038545333	7,33573E-11
Contig14-snap.170	0,090279986	1,83393E-10
Contig10-snap.464	0,165198215	2,7573E-09
Contig56-snap.97	0,040389945	2,7573E-09
Contig36-snap.191	0,221967444	2,2105E-08
Contig63-snap.66	0,226367696	2,68968E-08
Contig97-snap.102	0,149884178	6,34591E-08
Contig14-snap.131	0,178311295	9,9724E-08
Contig77-snap.99	0,079934383	1,1698E-07
Contig117-snap.28	0,208152097	1,17864E-07
Contig113-snap.53	0,247973868	1,36443E-07
Contig97-snap.4	0,147278628	1,45928E-07
Contig10-snap.379	0,054318219	1,45928E-07
Contig596-snap.1	0	4,02173E-07
Contig87-snap.105	0,174699817	4,65774E-07
Contig11-snap.483	0,028543182	9,35917E-07
Contig129-snap.4	0,254733393	1,25038E-06
Contig147-snap.12	0,066336651	1,63658E-06
Contig1-snap.88	0,063084964	2,10043E-06
Contig102-snap.20	0,143834334	2,52546E-06
Contig351-snap.3	0,083092956	2,72689E-06
Contig8-snap.30	0,006196976	4,8697E-06
Contig143-snap.4	0,237189163	9,43974E-06
Contig61-snap.159	0,029406523	1,26452E-05
Contig14-snap.382	0,224699684	1,69382E-05
Contig57-snap.107	0,031562165	1,87804E-05
Contig6-snap.122	0,12688206	3,25359E-05
Contig66-snap.69	0,097884891	5,78653E-05
Contig32-snap.276	0,147143937	8,44083E-05
Contig56-snap.180	0,179479379	8,54968E-05
Contig113-snap.52	0,333262293	0,000095418

Contig109-snap.22	0,206883429	9,54641E-05
Contig320-snap.3	0,132744133	9,54641E-05
Contig125-snap.27	0,240678638	9,63592E-05
Contig17-snap.49	0,232415791	0,00010668
Contig103-snap.29	0,103387953	0,00010668
Contig4-snap.211	0,091354525	0,00010668
Contig30-snap.226	0,055461483	0,00010668
Contig69-snap.22	0,005980327	0,00010668
Contig5-snap.274	0,228739908	0,000110519
Contig10-snap.436	0,051785787	0,000132014
Contig36-snap.257	0,006023259	0,000176584
Contig100-snap.48	0,354495692	0,000207246
Contig538-snap.2	0,103939073	0,000303722
Contig90-snap.13	0,288595358	0,000325322
Contig11-snap.43	0,254805796	0,000365236
Contig17-snap.264	0,156744856	0,000505174
Contig75-snap.112	0,013192301	0,000517447
Contig0-snap.381	0,064646228	0,000553292
Contig9-snap.313	0,201031015	0,000585007
Contig141-snap.25	0,135514581	0,000604639
Contig33-snap.23	0,382247773	0,000625135
Contig105-snap.26	0,045746019	0,000705992
Contig110-snap.55	0,025046451	0,000725377
Contig1-snap.299	0,148843693	0,000733087
Contig41-snap.114	0,182167816	0,000758377
Contig2-snap.318	0,060444295	0,000792405
Contig127-snap.54	0,079514953	0,000864763
Contig2-snap.275	0,09453681	0,000987442
Contig43-snap.58	0,292072817	0,00104124
Contig124-snap.25	0,084121594	0,0010463
Contig30-snap.268	0,346519873	0,00112257
Contig127-snap.25	0,290278575	0,00113623
Contig14-snap.198	0	0,00119696
Contig14-snap.67	0,136411767	0,00125167
Contig176-snap.15	0,337937885	0,00156484
Contig77-snap.126	0,235315784	0,00159015
Contig20-snap.87	0,165081459	0,00162149
Contig62-snap.7	0,149554166	0,00197536
Contig32-snap.157	0,077255422	0,00201161
Contig30-snap.201	0,221253165	0,00221731
Contig105-snap.6	0,276779692	0,00236551
Contig61-snap.89	0,337659253	0,00247332
Contig180-snap.9	0,020522354	0,00251249
Contig746-snap.1	0,230706411	0,0026016
Contig55-snap.82	0,198706366	0,00266865
Contig31-snap.216	0,4009516	0,00270048
Contig13-snap.374	0,342130826	0,0027383
Contig138-snap.6	0,012526177	0,00284796
Contig115-snap.3	0,390363481	0,00333557
Contig25-snap.92	0,233240464	0,00333557
Contig316-snap.1	0,061844967	0,00333557
Contig638-snap.1	0,223393637	0,00345627
Contig100-snap.5	0,351983572	0,00355141
Contig226-snap.2	0,133744364	0,00360474
Contig23-snap.196	0,19687475	0,00377964

Contig11-snap.474	0,274038405	0,00394852
Contig68-snap.24	0,218887276	0,00394852
Contig11-snap.260	0,404208061	0,00409407
Contig50-snap.44	0,395749656	0,00440543
Contig11-snap.210	0,116315882	0,0046836
Contig18-snap.93	0,23959837	0,00475155
Contig31-snap.69	0,406788275	0,00505112
Contig11-snap.183	0,185316083	0,00516887
Contig30-snap.188	0,086845807	0,00516887
Contig102-snap.8	0,117734578	0,00532036
Contig14-snap.349	0,036243598	0,00547608
Contig2-snap.317	0,067416241	0,00638273
Contig12-snap.221	0,254394608	0,00642965
Contig43-snap.22	0,276962009	0,00698537
Contig89-snap.78	0,156075934	0,00698537
Contig11-snap.402	0,201710767	0,00709599
Contig11-snap.150	0,313196501	0,00714069
Contig9-snap.131	0,015545922	0,00724048
Contig0-snap.344	0,443513523	0,00790813
Contig61-snap.10	0,159160032	0,00790813
Contig2-snap.327	0,403664886	0,00791615
Contig10-snap.289	0,091353259	0,00809759
Contig8-snap.280	0,437052956	0,00843321
Contig30-snap.63	0,290153855	0,00874019
Contig11-snap.7	0,168225697	0,00892375
Contig14-snap.267	0,177325264	0,00917801
Contig14-snap.68	0,255069093	0,00926488
Contig97-snap.37	0,214714273	0,00937072
Contig75-snap.2	0,309684268	0,00969665
Contig35-snap.117	0,259160904	0,00993981
Contig99-snap.45	0,199891634	0,00996932
Contig14-snap.384	0,016946252	0,0100429
Contig75-snap.45	0,128483945	0,0103225
Contig33-snap.111	0,090981057	0,0103225
Contig35-snap.18	0,296694482	0,01052
Contig10-snap.340	0,304746171	0,0105511
Contig5-snap.72	0,017931252	0,0106252
Contig56-snap.84	0,313781021	0,0107445
Contig104-snap.11	0,041949284	0,0107445
Contig1160-snap.1	0	0,012323
Contig5-snap.325	0,248281728	0,0124433
Contig43-snap.55	0,349024448	0,012506
Contig50-snap.120	0,193104006	0,012506
Contig10-snap.254	0,24399441	0,012591
Contig25-snap.48	0,045720025	0,0128824
Contig61-snap.126	0,219836066	0,0140299
Contig272-snap.1	0,139846883	0,0143238
Contig6-snap.268	0,396328879	0,0143824
Contig22-snap.178	0,220331856	0,0143824
Contig34-snap.10	0,046566943	0,0143824
Contig103-snap.59	0,276860281	0,0145896
Contig145-snap.29	0,397611117	0,0149171
Contig119-snap.50	0,019156529	0,0149171
Contig11-snap.241	0,212106391	0,0149967
Contig103-snap.51	0,335020202	0,0156301

Contig13-snap.56	0,174639281	0,0156301
Contig87-snap.122	0,301683981	0,0160403
Contig40-snap.66	0,043539541	0,0170466
Contig20-snap.196	0,063252223	0,0172997
Contig7-snap.348	0,039360436	0,0192332
Contig139-snap.33	0,234516283	0,0199627
Contig60-snap.12	0,047230087	0,0199857
Contig61-snap.81	0,23069042	0,0202204
Contig11-snap.296	0,053070355	0,0207815
Contig68-snap.73	0,203165874	0,0214741
Contig103-snap.99	0,29197161	0,0218902
Contig46-snap.15	0,121971839	0,0218902
Contig8-snap.184	0,090620421	0,0218902
Contig529-snap.1	0,188650784	0,0221346
Contig11-snap.151	0,086109129	0,0224133
Contig138-snap.5	0,24235101	0,0226078
Contig31-snap.142	0,185674809	0,0226078
Contig57-snap.69	0,099234804	0,0226078
Contig125-snap.44	0,33974636	0,0226978
Contig255-snap.2	0,028524591	0,0228273
Contig71-snap.30	0,112669583	0,0229579
Contig81-snap.40	0,02201911	0,0229579
Contig14-snap.47	0,375631512	0,0233194
Contig23-snap.104	0,222588351	0,0246983
Contig31-snap.62	0,128226824	0,0249605
Contig98-snap.66	0,436568517	0,0268217
Contig11-snap.319	0,30534455	0,0268217
Contig14-snap.63	0,159719238	0,0268217
Contig130-snap.24	0,060521015	0,0268217
Contig4-snap.213	0,182392713	0,0275784
Contig8-snap.141	0,117972295	0,0275784
Contig56-snap.69	0,179478135	0,0284166
Contig75-snap.4	0,177300683	0,0284317
Contig296-snap.2	0,034001919	0,0284782
Contig70-snap.8	0,158220671	0,0291303
Contig79-snap.18	0,217012492	0,0297596
Contig32-snap.76	0,032265138	0,0297596
Contig14-snap.86	0,35259893	0,0301426
Contig419-snap.1	0,234708175	0,0301426
Contig45-snap.121	0,171517775	0,0306335
Contig10-snap.456	0,239975662	0,0315463
Contig19-snap.51	0,065352218	0,0315463
Contig39-snap.128	0	0,0315463
Contig25-snap.107	0,149760597	0,0315887
Contig24-snap.271	0,461224163	0,0316623
Contig85-snap.22	0,032946522	0,0318677
Contig26-snap.36	0,180418602	0,0327456
Contig39-snap.102	0,372419366	0,0328329
Contig123-snap.1	0,101530142	0,0328329
Contig17-snap.252	0,023531384	0,0328329
Contig139-snap.55	0,165925784	0,0333581
Contig14-snap.33	0,025719208	0,0333581
Contig17-snap.112	0,245363065	0,0335256
Contig145-snap.30	0,408220348	0,0335616
Contig1-snap.82	0,226306511	0,0364589

Contig14-snap.357	0,069649171	0,0364589
Contig10-snap.373	0,048384877	0,0364589
Contig28-snap.217	0,374417559	0,0365082
Contig14-snap.311	0,3900227	0,0379509
Contig97-snap.126	0,178337252	0,0379509
Contig27-snap.188	0,105757362	0,0379509
Contig31-snap.323	0,429086376	0,039204
Contig26-snap.33	0,139935121	0,0394386
Contig47-snap.162	0,041426565	0,0394386
Contig26-snap.105	0,035200507	0,0396762
Contig69-snap.9	0	0,0396762
Contig59-snap.151	0,395831958	0,0399193
Contig23-snap.122	0,057827919	0,0409057
Contig68-snap.82	0,026065584	0,0414377
Contig145-snap.27	0,473931349	0,0420894
Contig30-snap.228	0,272855683	0,0420894
Contig14-snap.100	0,196356873	0,0422089
Contig49-snap.25	0,139910874	0,0422089
Contig18-snap.193	0	0,0422089
Contig144-snap.39	0,138325592	0,0427174
Contig71-snap.22	0,045734605	0,0429852
Contig32-snap.91	0,299299132	0,04327
Contig15-snap.71	0,505167594	0,0436495
Contig97-snap.107	0,346027835	0,0436495
Contig5-snap.67	0,280077889	0,0436495
Contig26-snap.72	0,27336681	0,0436495
Contig18-snap.260	0,158129671	0,0436495
Contig24-snap.174	0,075086117	0,0436495
Contig73-snap.30	0,044867357	0,0436495
Contig123-snap.25	0,196818809	0,0440327
Contig14-snap.199	0,233918845	0,044093
Contig140-snap.28	0,444649356	0,0451409
Contig126-snap.11	0,028468889	0,0451409
Contig10-snap.426	0,087819188	0,0471901
Contig17-snap.161	0,065427457	0,0478031
Contig14-snap.418	0,044978208	0,0478031
Contig17-snap.45	0,428022932	0,0485581
Contig31-snap.287	0,108101616	0,0485581
Contig8-snap.357	0,485051555	0,0487759
Contig627-snap.1	0,29613358	0,0490452
Contig100-snap.50	0,400163088	0,0493838
Contig0-snap.323	0,052822998	0,0493838

Supplementary Table 2: List of gene specific primers of *lsy-12* 3' and 5' RACE

CDNA END	PRIMER	OLIGO
3'	Co6GSP2	GCCGTGTTTGTCGCCGCAGTACGCGAGA
	Co6NGSP2	GAGGGCGCGAGCAGCAGCAATGAGGACG
	Co6.1GSP2	GAAATCGCGGCGGACACGGGCATCTCTC
	Co6.1NSP2	GGCGCTCGGGATGCTCGTCAAGAGCGAG
	Co6.2GSP2	GCTAAATTTTGGCATGGCCCCTCACTCG
	Co6.2NGSP2	GCGGCGGTATGCAGTCCACGCCGGGCAG
5'	Co6GSP1	GTACTTGAGGCAGAACTCGCAGATGTAC
	Co6NGSP1	GATAGGGCGCGGTGAACCACGTCTCCAT
	Co6.1GSP1	CCTCATATGCCGCCCACTGGTCCTGCGT
	Co6.1NGSP1	GCGTCGGCTCGTCACTGGGCGGCATCGA

Supplementary Table 3: List of primers used for as-*eud-1* transcript identification

Primer	Oligo
Co10F3	GGAAGACTTCCGCAAGAAGCATGC
Co10R6	CTCGAGTGTTCATTCCTCAGGACATTG
Co10F4	AGGGCCGAGTGGGCTACAAGGG
Co10R5	CCTACTACATCTCCACGCAGTGATC
CoasFn	GAAGAACAAAACACACGGAGAAG
Co10asRn	ACTACCGAAAGACGAGACTACAATG

Supplementary Table 4: List of antibodies used in this study

Antibody	Host	Type	Company	Dilution	Cat. #
Alpha tubulin	Mouse	mAb	SIGMA ALDRICH	1:100	F22168
H3K4me3	Mouse	mAb	Diagenenode	1:1000	C15200152
H3K4me2	Rabbit	pAb	Diagenenode	1:1000	C15410035
H3K4me1	Mouse	mAb	Diagenenode	1:1000	Mab-150-050
H3K9ac	Mouse	mAb	Diagenenode	1:1000	Mab-185-050
H3K27ac	Mouse	mAb	Diagenenode	1:1000	C15200184
H2Bub	Mouse	mAb	Active Motif	1:1000	39623, 39624
H2Aub	Mouse	mAb	Millipore	1:2000	05-678
H4K20me1	Rabbit	pAb	Abcam	1:1000	9051
