# Supplementary material for: Chromatin remodelling and antisense-mediated up-regulation of the developmental switch gene eud-1 control predatory feeding plasticity
Source: Nat Commun. 2016 Aug 4;7:12337. doi: 10.1038/ncomms12337 (PMC4976200; doi:10.1038/ncomms12337)
Supplement: Supplementary Data 1 — Differentially expressed genes between wild type and Ppa-lsy-12 mutant animals (blue, up-regulated; green, down regulated). [file ncomms12337-s3.docx]

**Supplementary Data 1: Differentially expressed genes between wild type and *Ppa-lsy-12* mutant animals (blue, up-regulated; green, down regulated).**

| **P. pacificus Gene ID (version Hybrid1)** | **Expression foldchange** | **P (FDR corrected)** |
| --- | --- | --- |
| Contig41-snap.23 | 13,29000737 | 1,84319E-07 |
| Contig22-snap.173 | 9,101661929 | 9,63592E-05 |
| Contig11-snap.172 | 6,027375725 | 0,000110313 |
| Contig70-snap.1 | 12,39485939 | 0,000128043 |
| Contig12-snap.157 | 3,697320223 | 0,000149273 |
| Contig106-snap.25 | 4,775295463 | 0,000300751 |
| Contig10-snap.353 | 11,18109827 | 0,000317931 |
| Contig41-snap.82 | 7,096117999 | 0,000919555 |
| Contig77-snap.8 | 65,96916778 | 0,00100054 |
| Contig143-snap.16 | 16,83556227 | 0,00105018 |
| Contig4-snap.53 | 37,7475211 | 0,00123591 |
| Contig22-snap.106 | 3,599433838 | 0,00179514 |
| Contig52-snap.11 | 4,42065455 | 0,00284796 |
| Contig29-snap.5 | 61,43291773 | 0,00333557 |
| Contig30-snap.191 | 11,6756422 | 0,00333557 |
| Contig65-snap.25 | inf | 0,00409407 |
| Contig80-snap.15 | 3,577619607 | 0,0046836 |
| Contig81-snap.24 | 14,3205004 | 0,00530466 |
| Contig11-snap.371 | 2,536642133 | 0,00549032 |
| Contig60-snap.86 | 87,23891972 | 0,00709599 |
| Contig43-snap.76 | 5,296245508 | 0,00714069 |
| Contig41-snap.5 | 4,873269472 | 0,00780334 |
| Contig70-snap.59 | 3,294820797 | 0,00829626 |
| Contig5-snap.322 | 79,30225582 | 0,00843321 |
| Contig14-snap.271 | 3,385760678 | 0,00917801 |
| Contig50-snap.180 | 2,637900229 | 0,00969665 |
| Contig18-snap.207 | 78,60504474 | 0,0103225 |
| Contig21-snap.203 | 5,691977662 | 0,0107445 |
| Contig114-snap.47 | 3,607451503 | 0,01173 |
| Contig70-snap.44 | 21,23570353 | 0,011961 |
| Contig5-snap.109 | 73,6753447 | 0,0122673 |
| Contig15-snap.100 | 26,78809343 | 0,012506 |
| Contig2-snap.143 | 39,36795862 | 0,0133108 |
| Contig43-snap.100 | 2,800452581 | 0,0143238 |
| Contig122-snap.33 | 12,62892558 | 0,0143824 |
| Contig11-snap.54 | 8,836162803 | 0,0170202 |
| Contig45-snap.65 | 10,71500041 | 0,0205952 |
| Contig0-snap.107 | 4,994949181 | 0,0206185 |
| Contig45-snap.142 | inf | 0,0206552 |
| Contig1-snap.244 | 4,337248188 | 0,0221536 |
| Contig45-snap.89 | 12,50765998 | 0,0224134 |
| Contig125-snap.20 | 3,921566323 | 0,0224164 |
| Contig13-snap.199 | 2,791498924 | 0,0228273 |
| Contig165-snap.14 | inf | 0,0268869 |
| Contig91-snap.44 | 3,577669204 | 0,0272269 |
| Contig13-snap.239 | 7,533132996 | 0,0280861 |
| Contig36-snap.166 | 2,722458392 | 0,0282879 |
| Contig93-snap.38 | inf | 0,0301426 |
| Contig65-snap.26 | 8,275520184 | 0,0314173 |
| Contig23-snap.131 | 9,258317253 | 0,0328285 |
| Contig7-snap.59 | 3,557761992 | 0,0328329 |
| Contig49-snap.90 | 3,471285437 | 0,0335256 |
| Contig0-snap.498 | 2,38574525 | 0,03497 |
| Contig5-snap.298 | 2,253129132 | 0,0364589 |
| Contig29-snap.28 | 4,126704113 | 0,0379509 |
| Contig12-snap.10 | inf | 0,0391579 |
| Contig99-snap.10 | 2,124995815 | 0,039204 |
| Contig155-snap.3 | 47,83915494 | 0,0394386 |
| Contig12-snap.416 | 3,1659753 | 0,0409838 |
| Contig3-snap.167 | 2,16899992 | 0,0409838 |
| Contig125-snap.78 | 2,846699088 | 0,0411583 |
| Contig11-snap.380 | 2,20643921 | 0,0434612 |
| Contig43-snap.120 | 2,218062472 | 0,0438765 |
| Contig78-snap.48 | inf | 0,0451409 |
| Contig23-snap.197 | 54,92039132 | 0,0451409 |
| Contig111-snap.8 | 45,22943284 | 0,0451409 |
| Contig106-snap.1 | 18,20698332 | 0,0487759 |
| Contig0-snap.648 | 3,022369399 | 0,0490452 |
| Contig116-snap.30 | 3,456471428 | 0,0491213 |
| Contig1-snap.120 | inf | 0,0493838 |
| Contig28-snap.265 | 0,129880793 | 0 |
| Contig97-snap.101 | 0,076369479 | 1,7466E-12 |
| Contig56-snap.93 | 0,038545333 | 7,33573E-11 |
| Contig14-snap.170 | 0,090279986 | 1,83393E-10 |
| Contig10-snap.464 | 0,165198215 | 2,7573E-09 |
| Contig56-snap.97 | 0,040389945 | 2,7573E-09 |
| Contig36-snap.191 | 0,221967444 | 2,2105E-08 |
| Contig63-snap.66 | 0,226367696 | 2,68968E-08 |
| Contig97-snap.102 | 0,149884178 | 6,34591E-08 |
| Contig14-snap.131 | 0,178311295 | 9,9724E-08 |
| Contig77-snap.99 | 0,079934383 | 1,1698E-07 |
| Contig117-snap.28 | 0,208152097 | 1,17864E-07 |
| Contig113-snap.53 | 0,247973868 | 1,36443E-07 |
| Contig97-snap.4 | 0,147278628 | 1,45928E-07 |
| Contig10-snap.379 | 0,054318219 | 1,45928E-07 |
| Contig596-snap.1 | 0 | 4,02173E-07 |
| Contig87-snap.105 | 0,174699817 | 4,65774E-07 |
| Contig11-snap.483 | 0,028543182 | 9,35917E-07 |
| Contig129-snap.4 | 0,254733393 | 1,25038E-06 |
| Contig147-snap.12 | 0,066336651 | 1,63658E-06 |
| Contig1-snap.88 | 0,063084964 | 2,10043E-06 |
| Contig102-snap.20 | 0,143834334 | 2,52546E-06 |
| Contig351-snap.3 | 0,083092956 | 2,72689E-06 |
| Contig8-snap.30 | 0,006196976 | 4,8697E-06 |
| Contig143-snap.4 | 0,237189163 | 9,43974E-06 |
| Contig61-snap.159 | 0,029406523 | 1,26452E-05 |
| Contig14-snap.382 | 0,224699684 | 1,69382E-05 |
| Contig57-snap.107 | 0,031562165 | 1,87804E-05 |
| Contig6-snap.122 | 0,12688206 | 3,25359E-05 |
| Contig66-snap.69 | 0,097884891 | 5,78653E-05 |
| Contig32-snap.276 | 0,147143937 | 8,44083E-05 |
| Contig56-snap.180 | 0,179479379 | 8,54968E-05 |
| Contig113-snap.52 | 0,333262293 | 0,000095418 |
| Contig109-snap.22 | 0,206883429 | 9,54641E-05 |
| Contig320-snap.3 | 0,132744133 | 9,54641E-05 |
| Contig125-snap.27 | 0,240678638 | 9,63592E-05 |
| Contig17-snap.49 | 0,232415791 | 0,00010668 |
| Contig103-snap.29 | 0,103387953 | 0,00010668 |
| Contig4-snap.211 | 0,091354525 | 0,00010668 |
| Contig30-snap.226 | 0,055461483 | 0,00010668 |
| Contig69-snap.22 | 0,005980327 | 0,00010668 |
| Contig5-snap.274 | 0,228739908 | 0,000110519 |
| Contig10-snap.436 | 0,051785787 | 0,000132014 |
| Contig36-snap.257 | 0,006023259 | 0,000176584 |
| Contig100-snap.48 | 0,354495692 | 0,000207246 |
| Contig538-snap.2 | 0,103939073 | 0,000303722 |
| Contig90-snap.13 | 0,288595358 | 0,000325322 |
| Contig11-snap.43 | 0,254805796 | 0,000365236 |
| Contig17-snap.264 | 0,156744856 | 0,000505174 |
| Contig75-snap.112 | 0,013192301 | 0,000517447 |
| Contig0-snap.381 | 0,064646228 | 0,000553292 |
| Contig9-snap.313 | 0,201031015 | 0,000585007 |
| Contig141-snap.25 | 0,135514581 | 0,000604639 |
| Contig33-snap.23 | 0,382247773 | 0,000625135 |
| Contig105-snap.26 | 0,045746019 | 0,000705992 |
| Contig110-snap.55 | 0,025046451 | 0,000725377 |
| Contig1-snap.299 | 0,148843693 | 0,000733087 |
| Contig41-snap.114 | 0,182167816 | 0,000758377 |
| Contig2-snap.318 | 0,060444295 | 0,000792405 |
| Contig127-snap.54 | 0,079514953 | 0,000864763 |
| Contig2-snap.275 | 0,09453681 | 0,000987442 |
| Contig43-snap.58 | 0,292072817 | 0,00104124 |
| Contig124-snap.25 | 0,084121594 | 0,0010463 |
| Contig30-snap.268 | 0,346519873 | 0,00112257 |
| Contig127-snap.25 | 0,290278575 | 0,00113623 |
| Contig14-snap.198 | 0 | 0,00119696 |
| Contig14-snap.67 | 0,136411767 | 0,00125167 |
| Contig176-snap.15 | 0,337937885 | 0,00156484 |
| Contig77-snap.126 | 0,235315784 | 0,00159015 |
| Contig20-snap.87 | 0,165081459 | 0,00162149 |
| Contig62-snap.7 | 0,149554166 | 0,00197536 |
| Contig32-snap.157 | 0,077255422 | 0,00201161 |
| Contig30-snap.201 | 0,221253165 | 0,00221731 |
| Contig105-snap.6 | 0,276779692 | 0,00236551 |
| Contig61-snap.89 | 0,337659253 | 0,00247332 |
| Contig180-snap.9 | 0,020522354 | 0,00251249 |
| Contig746-snap.1 | 0,230706411 | 0,0026016 |
| Contig55-snap.82 | 0,198706366 | 0,00266865 |
| Contig31-snap.216 | 0,4009516 | 0,00270048 |
| Contig13-snap.374 | 0,342130826 | 0,0027383 |
| Contig138-snap.6 | 0,012526177 | 0,00284796 |
| Contig115-snap.3 | 0,390363481 | 0,00333557 |
| Contig25-snap.92 | 0,233240464 | 0,00333557 |
| Contig316-snap.1 | 0,061844967 | 0,00333557 |
| Contig638-snap.1 | 0,223393637 | 0,00345627 |
| Contig100-snap.5 | 0,351983572 | 0,00355141 |
| Contig226-snap.2 | 0,133744364 | 0,00360474 |
| Contig23-snap.196 | 0,19687475 | 0,00377964 |
| Contig11-snap.474 | 0,274038405 | 0,00394852 |
| Contig68-snap.24 | 0,218887276 | 0,00394852 |
| Contig11-snap.260 | 0,404208061 | 0,00409407 |
| Contig50-snap.44 | 0,395749656 | 0,00440543 |
| Contig11-snap.210 | 0,116315882 | 0,0046836 |
| Contig18-snap.93 | 0,23959837 | 0,00475155 |
| Contig31-snap.69 | 0,406788275 | 0,00505112 |
| Contig11-snap.183 | 0,185316083 | 0,00516887 |
| Contig30-snap.188 | 0,086845807 | 0,00516887 |
| Contig102-snap.8 | 0,117734578 | 0,00532036 |
| Contig14-snap.349 | 0,036243598 | 0,00547608 |
| Contig2-snap.317 | 0,067416241 | 0,00638273 |
| Contig12-snap.221 | 0,254394608 | 0,00642965 |
| Contig43-snap.22 | 0,276962009 | 0,00698537 |
| Contig89-snap.78 | 0,156075934 | 0,00698537 |
| Contig11-snap.402 | 0,201710767 | 0,00709599 |
| Contig11-snap.150 | 0,313196501 | 0,00714069 |
| Contig9-snap.131 | 0,015545922 | 0,00724048 |
| Contig0-snap.344 | 0,443513523 | 0,00790813 |
| Contig61-snap.10 | 0,159160032 | 0,00790813 |
| Contig2-snap.327 | 0,403664886 | 0,00791615 |
| Contig10-snap.289 | 0,091353259 | 0,00809759 |
| Contig8-snap.280 | 0,437052956 | 0,00843321 |
| Contig30-snap.63 | 0,290153855 | 0,00874019 |
| Contig11-snap.7 | 0,168225697 | 0,00892375 |
| Contig14-snap.267 | 0,177325264 | 0,00917801 |
| Contig14-snap.68 | 0,255069093 | 0,00926488 |
| Contig97-snap.37 | 0,214714273 | 0,00937072 |
| Contig75-snap.2 | 0,309684268 | 0,00969665 |
| Contig35-snap.117 | 0,259160904 | 0,00993981 |
| Contig99-snap.45 | 0,199891634 | 0,00996932 |
| Contig14-snap.384 | 0,016946252 | 0,0100429 |
| Contig75-snap.45 | 0,128483945 | 0,0103225 |
| Contig33-snap.111 | 0,090981057 | 0,0103225 |
| Contig35-snap.18 | 0,296694482 | 0,01052 |
| Contig10-snap.340 | 0,304746171 | 0,0105511 |
| Contig5-snap.72 | 0,017931252 | 0,0106252 |
| Contig56-snap.84 | 0,313781021 | 0,0107445 |
| Contig104-snap.11 | 0,041949284 | 0,0107445 |
| Contig1160-snap.1 | 0 | 0,012323 |
| Contig5-snap.325 | 0,248281728 | 0,0124433 |
| Contig43-snap.55 | 0,349024448 | 0,012506 |
| Contig50-snap.120 | 0,193104006 | 0,012506 |
| Contig10-snap.254 | 0,24399441 | 0,012591 |
| Contig25-snap.48 | 0,045720025 | 0,0128824 |
| Contig61-snap.126 | 0,219836066 | 0,0140299 |
| Contig272-snap.1 | 0,139846883 | 0,0143238 |
| Contig6-snap.268 | 0,396328879 | 0,0143824 |
| Contig22-snap.178 | 0,220331856 | 0,0143824 |
| Contig34-snap.10 | 0,046566943 | 0,0143824 |
| Contig103-snap.59 | 0,276860281 | 0,0145896 |
| Contig145-snap.29 | 0,397611117 | 0,0149171 |
| Contig119-snap.50 | 0,019156529 | 0,0149171 |
| Contig11-snap.241 | 0,212106391 | 0,0149967 |
| Contig103-snap.51 | 0,335020202 | 0,0156301 |
| Contig13-snap.56 | 0,174639281 | 0,0156301 |
| Contig87-snap.122 | 0,301683981 | 0,0160403 |
| Contig40-snap.66 | 0,043539541 | 0,0170466 |
| Contig20-snap.196 | 0,063252223 | 0,0172997 |
| Contig7-snap.348 | 0,039360436 | 0,0192332 |
| Contig139-snap.33 | 0,234516283 | 0,0199627 |
| Contig60-snap.12 | 0,047230087 | 0,0199857 |
| Contig61-snap.81 | 0,23069042 | 0,0202204 |
| Contig11-snap.296 | 0,053070355 | 0,0207815 |
| Contig68-snap.73 | 0,203165874 | 0,0214741 |
| Contig103-snap.99 | 0,29197161 | 0,0218902 |
| Contig46-snap.15 | 0,121971839 | 0,0218902 |
| Contig8-snap.184 | 0,090620421 | 0,0218902 |
| Contig529-snap.1 | 0,188650784 | 0,0221346 |
| Contig11-snap.151 | 0,086109129 | 0,0224133 |
| Contig138-snap.5 | 0,24235101 | 0,0226078 |
| Contig31-snap.142 | 0,185674809 | 0,0226078 |
| Contig57-snap.69 | 0,099234804 | 0,0226078 |
| Contig125-snap.44 | 0,33974636 | 0,0226978 |
| Contig255-snap.2 | 0,028524591 | 0,0228273 |
| Contig71-snap.30 | 0,112669583 | 0,0229579 |
| Contig81-snap.40 | 0,02201911 | 0,0229579 |
| Contig14-snap.47 | 0,375631512 | 0,0233194 |
| Contig23-snap.104 | 0,222588351 | 0,0246983 |
| Contig31-snap.62 | 0,128226824 | 0,0249605 |
| Contig98-snap.66 | 0,436568517 | 0,0268217 |
| Contig11-snap.319 | 0,30534455 | 0,0268217 |
| Contig14-snap.63 | 0,159719238 | 0,0268217 |
| Contig130-snap.24 | 0,060521015 | 0,0268217 |
| Contig4-snap.213 | 0,182392713 | 0,0275784 |
| Contig8-snap.141 | 0,117972295 | 0,0275784 |
| Contig56-snap.69 | 0,179478135 | 0,0284166 |
| Contig75-snap.4 | 0,177300683 | 0,0284317 |
| Contig296-snap.2 | 0,034001919 | 0,0284782 |
| Contig70-snap.8 | 0,158220671 | 0,0291303 |
| Contig79-snap.18 | 0,217012492 | 0,0297596 |
| Contig32-snap.76 | 0,032265138 | 0,0297596 |
| Contig14-snap.86 | 0,35259893 | 0,0301426 |
| Contig419-snap.1 | 0,234708175 | 0,0301426 |
| Contig45-snap.121 | 0,171517775 | 0,0306335 |
| Contig10-snap.456 | 0,239975662 | 0,0315463 |
| Contig19-snap.51 | 0,065352218 | 0,0315463 |
| Contig39-snap.128 | 0 | 0,0315463 |
| Contig25-snap.107 | 0,149760597 | 0,0315887 |
| Contig24-snap.271 | 0,461224163 | 0,0316623 |
| Contig85-snap.22 | 0,032946522 | 0,0318677 |
| Contig26-snap.36 | 0,180418602 | 0,0327456 |
| Contig39-snap.102 | 0,372419366 | 0,0328329 |
| Contig123-snap.1 | 0,101530142 | 0,0328329 |
| Contig17-snap.252 | 0,023531384 | 0,0328329 |
| Contig139-snap.55 | 0,165925784 | 0,0333581 |
| Contig14-snap.33 | 0,025719208 | 0,0333581 |
| Contig17-snap.112 | 0,245363065 | 0,0335256 |
| Contig145-snap.30 | 0,408220348 | 0,0335616 |
| Contig1-snap.82 | 0,226306511 | 0,0364589 |
| Contig14-snap.357 | 0,069649171 | 0,0364589 |
| Contig10-snap.373 | 0,048384877 | 0,0364589 |
| Contig28-snap.217 | 0,374417559 | 0,0365082 |
| Contig14-snap.311 | 0,3900227 | 0,0379509 |
| Contig97-snap.126 | 0,178337252 | 0,0379509 |
| Contig27-snap.188 | 0,105757362 | 0,0379509 |
| Contig31-snap.323 | 0,429086376 | 0,039204 |
| Contig26-snap.33 | 0,139935121 | 0,0394386 |
| Contig47-snap.162 | 0,041426565 | 0,0394386 |
| Contig26-snap.105 | 0,035200507 | 0,0396762 |
| Contig69-snap.9 | 0 | 0,0396762 |
| Contig59-snap.151 | 0,395831958 | 0,0399193 |
| Contig23-snap.122 | 0,057827919 | 0,0409057 |
| Contig68-snap.82 | 0,026065584 | 0,0414377 |
| Contig145-snap.27 | 0,473931349 | 0,0420894 |
| Contig30-snap.228 | 0,272855683 | 0,0420894 |
| Contig14-snap.100 | 0,196356873 | 0,0422089 |
| Contig49-snap.25 | 0,139910874 | 0,0422089 |
| Contig18-snap.193 | 0 | 0,0422089 |
| Contig144-snap.39 | 0,138325592 | 0,0427174 |
| Contig71-snap.22 | 0,045734605 | 0,0429852 |
| Contig32-snap.91 | 0,299299132 | 0,04327 |
| Contig15-snap.71 | 0,505167594 | 0,0436495 |
| Contig97-snap.107 | 0,346027835 | 0,0436495 |
| Contig5-snap.67 | 0,280077889 | 0,0436495 |
| Contig26-snap.72 | 0,27336681 | 0,0436495 |
| Contig18-snap.260 | 0,158129671 | 0,0436495 |
| Contig24-snap.174 | 0,075086117 | 0,0436495 |
| Contig73-snap.30 | 0,044867357 | 0,0436495 |
| Contig123-snap.25 | 0,196818809 | 0,0440327 |
| Contig14-snap.199 | 0,233918845 | 0,044093 |
| Contig140-snap.28 | 0,444649356 | 0,0451409 |
| Contig126-snap.11 | 0,028468889 | 0,0451409 |
| Contig10-snap.426 | 0,087819188 | 0,0471901 |
| Contig17-snap.161 | 0,065427457 | 0,0478031 |
| Contig14-snap.418 | 0,044978208 | 0,0478031 |
| Contig17-snap.45 | 0,428022932 | 0,0485581 |
| Contig31-snap.287 | 0,108101616 | 0,0485581 |
| Contig8-snap.357 | 0,485051555 | 0,0487759 |
| Contig627-snap.1 | 0,29613358 | 0,0490452 |
| Contig100-snap.50 | 0,400163088 | 0,0493838 |
| Contig0-snap.323 | 0,052822998 | 0,0493838 |
